# Avian malaria and the overlooked metabolic pathways underlying mosquito*–Plasmodium* interactions

**DOI:** 10.1098/rsos.250542

**Published:** 2025-10-22

**Authors:** Luz Garcia-Longoria, Arnaud Berthomieu, O. Hellgren, Ana Rivero

**Affiliations:** ^1^ Universidad de Extremadura Facultad de Ciencias, Badajoz, Spain; ^2^ MIVEGEC, (University of Montpellier, CNRS, IRD), Montpellier, France; ^3^ Evolutionary Ecology and Infection Biology, Department of Biology, Lund University, Lund, Sweden

**Keywords:** *Culex quinquefasciatus*, RNA-seq, transcriptomic, avian malaria, *Plasmodium relictum*

## Abstract

Avian malaria parasites pose a significant threat to conservation, affecting populations worldwide. Despite this, our understanding of factors influencing the transmission of avian *Plasmodium* parasites by vectors is limited to the study of mosquito immune responses. However, the complex life cycle of *Plasmodium* within the vector suggests that non-immune physiological and metabolic pathways may play equally, if not more, crucial roles in determining whether the parasite completes its development and successfully transmits to the next host. We review some of these pathways, uncovering a fragmented and contradictory body of knowledge. Through transcriptomic analysis of infected and uninfected mosquitoes at various stages of infection, we identify differential expression of numerous metabolic pathways that are essential for *Plasmodium* development. These include genes involved in meeting the parasite’s energetic needs, digestive enzymes facilitating midgut barrier traversal, and salivary enzymes enabling blood meal uptake, among others. This suggests that the parasite has evolved mechanisms to modulate these pathways, thereby enhancing and prolonging infection and transmission. Our findings emphasize the need for a broader, integrative approach to better understand the reciprocal selective pressures between malaria parasites and their vectors and to find novel targets for controlling parasite transmission and ultimately improving malaria control strategies.

## 1. Introduction

Although malaria parasites (*Plasmodium* spp.) are most commonly associated with humans, they also infect hundreds of other terrestrial vertebrate species, including non-human primates, ungulates, rodents, bats, lizards and birds. Among these, avian malaria parasites are the most widespread, prevalent and diverse of all malaria parasites known to date [1]. Avian *Plasmodium* has been central to bird conservation research since the early twentieth century, when the accidental introduction of *Plasmodium relictum* to the Hawaiian Islands resulted in the decline and extinction of several endemic bird species [2,3]. Avian malaria is transmitted by Culicine mosquitoes, with species from the *Culex pipiens* complex serving as the dominant species across many regions [4]. As climate change expands the range of mosquito vectors, the occurrence, distribution and intensity of avian malaria infections are increasing globally [5], prompting the IUCN to classify *P. relictum* as one of the world’s worst invasive species [6].

Understanding how avian malaria parasites are transmitted is crucial for predicting their epidemiological and evolutionary dynamics, as well as for developing effective conservation strategies. The ability of mosquitoes to host and transmit *Plasmodium* is the outcome of complex co-evolutionary processes, where parasites and vectors engage in reciprocal adaptations that shape their interactions. Mosquito immune responses have been extensively studied as a crucial factor determining the success of infections resulting in significant advances in our understanding of the cellular and humoral effectors and upstream immune pathways that lead to the lysis, melanization or phagocytosis of an invading pathogen [7,8]. Interestingly, the results of studies comparing the immune transcriptome of *Plasmodium*-infected and uninfected mosquitoes strongly suggest that the parasite may be able to manipulate the mosquito immune response to its own advantage [9]. However, the complex life cycle of *Plasmodium* within the vector suggests that non-immune physiological and metabolic pathways may play equally, if not more, crucial roles in determining whether the parasite completes its development and successfully transmits to the next host.

To complete its life cycle within the mosquito, *Plasmodium* must undergo a series of highly coordinated developmental transitions, shifting between haploid, diploid and syncytial forms, invading different mosquito tissues, and overcoming major physiological barriers [10]. The journey begins when a mosquito takes an infected blood meal, ingesting male and female gametocytes. In the midgut, the gametocytes differentiate into gametes and fuse to form a zygote, which undergoes meiosis and develops into a motile ookinete [11,12]. The ookinete must then traverse both the peritrophic matrix—a protective chitinous barrier—and the midgut epithelium before attaching to the basal lamina and developing into an oocyst. Over the course of several days, the oocyst undergoes multiple rounds of nuclear division, producing thousands of sporozoites, which are eventually released into the haemolymph and migrate to the salivary glands, ready for transmission in the mosquito’s next blood meal.

At each stage, *Plasmodium* interacts with mosquito metabolic pathways that are crucial for its development. Gametocyte activation, for example, is triggered by environmental changes such as a drop in temperature and an increase in pH [11], but also by xanthurenic acid (XA), a mosquito-derived metabolite involved in eye pigment synthesis [13]. Digestive enzymes, including trypsins and carboxypeptidases, facilitate blood digestion but also influence parasite survival. Trypsin, in particular, is required to activate a parasite-secreted chitinase that enables ookinetes to penetrate the peritrophic matrix [14,15]. Sporozoite production is metabolically demanding, and there is increasing evidence that *Plasmodium* relies on mosquito lipid [16,17] and sugar [18,19] transport proteins, as well as hormonal and enzymatic pathways [20,21], to support oocyst development.

Despite their potential significance, the role of these mosquito metabolic pathways in *Plasmodium* development remains poorly characterized, as does our understanding of whether the parasite has evolved mechanisms to modulate these pathways, thereby enhancing and prolonging infection and transmission. Here, we review some of these pathways, uncovering a fragmented and at times contradictory body of knowledge. For instance, glucose transporter (GLUT) genes show highly inconsistent patterns across studies: some have been reported with higher mRNA abundance in Anopheles species infected with different Plasmodium spp., whereas other studies have found lower abundance under comparable conditions (see Table 2). Such variability underscores that mosquito–Plasmodium interactions are not universally conserved, even for core metabolic pathways, and may depend strongly on the vector–parasite combination. Most published studies have examined mosquito physiological factors in isolation, often using *Plasmodium*-mosquito combinations that do not occur in nature, leading to inconsistencies in the conclusions (tables 1–5). Here, we investigate the interaction between the most prevalent and widespread avian malaria parasite in Europe, *P. relictum* (pSGS1), and its main natural vector in the field, the mosquito *Culex pipiens*. Through comprehensive transcriptomic analysis of infected and uninfected mosquitoes at various stages of infection, we identify differential expression of numerous physiologic and metabolic pathways that have been reported as crucial or potentially crucial for *Plasmodium* development. We discuss to what extent this may be evidence for parasite adaptive manipulation of its vector or simply the mosquito’s response to the infection. Our focus on adaptive manipulation stems from the observation that infection-induced changes in the vector’s internal environment differ from pre-existing conditions in uninfected individuals, suggesting that parasites may actively shape their host’s physiology to enhance transmission. To our knowledge, this is the first comprehensive study focused on physio-metabolic responses to infection in a mosquito-*Plasmodium* system. By deepening our understanding of mosquito-*Plasmodium* interactions beyond immunity, we can better comprehend the selective pressures shaping their co-evolution and identify potential targets for disrupting parasite development within the vector.

**Table 1.** Role of non-immune physiological factors previously reported as being putatively involved in *Plasmodium* invasion and survival in the mosquito midgut. Natural mosquito/Plasmodium combinations are in bold. MQ = mosquito, PL = *Plasmodium*, Ag = *Anopheles gambiae*, Ac = *Anopheles coluzzi*, As = *Anopheles stephensi*, Aa= Aedes aegypti, Pf = *Plasmodium falciparum* (human), Pb = *Plasmodium berguei* (rodent), Pg = *Plasmodium gallinaceum* (avian), INFM = experimentally infected mosquitoes, KDM = knock-down mosquitoes, XA = Xanthurenic Acid.

molecule	function	experimental results	MQ/PL combination	references
3-hydroxykynurenine (HKT)	tryptophan catabolism and synthesis of eye pigment precursor (XA) in MQ. XA is essential for PL exflagellation. 3HK also has a negative influence on peritrophic matrix integrity.	upregulated expression in INFM	As/Pb	[22]
		parasite load increases in KDM	As/Pb	[13]
aminopeptidase N (APN)	digestive enzyme in MQ. Ligand for midgut invasion in PL.	parasite load decreases in KDM	Ag/Pf, Ag/Pb	[23]
carboxypeptidase A (CPA)	digestive enzyme in MQ. Favours PL oocyst development (unknown mechanism).	upregulated expression in INFM	As/Pb	[24]
carboxypeptidase B (CPB)	digestive enzyme in MQ. Favours PL oocyst development (may provide lysine and arginine for the parasite)	upregulated expression in INFM	Ag/Pf	[25]
		upregulated expression in INFM	As/Pf	[26]
D7 long salivary proteins	component of MQ saliva, re-ingested by the MQ during the blood meal. Facilitates PL colonization of the midgut (unknown mechanism)	upregulated expression in INFM	Ag/Pf	[27]
		parasite load decreases in KDM	Aa/Pg	[28]
fibrinogen-related protein 1 (FREP1)	pattern recognition receptor in MQ. Anchors PL ookinetes to the peritrophic matrix, facilitates midgut invasion	parasite load decreases in KDM	Ag/Pf	[29]
mucin	main component of the glycocalyx protecting the MQ midgut.	parasite load decreases in KDM	Ag/Pf, Ag/Pb	[30]
		parasite load decreases in KDM*	Aa/Pg	[31]
		parasite load decreases in KDM*	Pb/As	[32]
plasmodium receptor protein (P47Rec)	role in the cytosqueleton of MQ midgut cells. Serves as a receptor for P47, a surface protein of PL that is involved in immune evasion.	parasite load decreases in KDM	Ag/Pf	[33]
peritrophins	components of the peritrophic matrix in MQ. Barrier to PL infection.	parasite load increases in KDM	Ag/Pf	[34]
trypsin	digestive enzyme in MQ. Activation of PL chitinase for the traversal of the peritrophic matrix. May destroy PL oocysts in midgut	upregulated expression in INFM	Ag/Pf	[35]

**Table 2.** Role of non-immune physiological proteins previously reported as being putatively involved in *Plasmodium* energetic metabolism in the mosquito. Natural mosquito/Plasmodium combinations are in bold. MQ = mosquito, PL = *Plasmodium*, Ag = *Anopheles gambiae*, Ac = *Anopheles coluzzi*, As = *Anopheles stephensi*, Aa= Aedes aegypti, Pf = *Plasmodium falciparum* (human), Pb = *Plasmodium berguei* (rodent), Py = *Plasmodium yoelii* (rodent), Pg = *Plasmodium gallinaceum* (avian), INFM = experimentally infected mosquitoes, KDM = knock-down mosquitoes.

molecule	function	experimental results	MQ/PL combination	references
adipokinetic hormone (AKH)	mobilization of MQs lipid energy reserves. Source of lipids for energy and membrane neogenesis in PL	upregulated expression in INFM	Ag/Pf	[21]
		parasite load decreases in KDM	Ag/Pf	[21]
AMP-activated kinase (AMPK)	regulates ATP consumption and production. Stimulates gluconeogenesis	downregulated expression in INFM	Ag/Pf	[36]
		overexpression decreases parasite load	As/Pf	[37]
apolipophorins I and II (Apo I, Apo II)	components of lipophorin, lipid transport molecule in MQ. Source of lipids for developing oocysts in PL. Speeds up PL sporozoite development. Reduces the parasite-killing efficiency of MQ TEP1	parasite load decreases in KDM	Aa/Pg	[38]
		upregulated expression in INFM	Aa/Pg	[39]
		parasite load decreases in KDM	Ag/Pf	[40]
apolipophorin III (Apo III)	lipid transport, and immunity in MQ. Source of lipids for developing PL oocysts	upregulated expression in INFM	Aa/Pg	[41]
		parasite load decreases in KDM	Aa/Pg	[41]
glucose transporters (GLUT)	glucose transport in MQ. Source of glucose for PL development	upregulated in INFM	Ac/Pb	[42]
		downregulated in INFM	As/Pb	[43]
		upregulated in INFM	Ag/Pf	[36]
low density lipophorin receptors (LDLRs)	lipophorin receptors in MQ. Source of lipids for developing PL oocysts	upregulated expression in INFM	Aa/Pg	[38]
pantothenate kinase (PanK)	transforms pantothenate into coenzyme A (coA), a precursor of acetyl-coA in MQ. PL needs to obtain pantothenate from MQ to produce its own coA. PanK depletes MQ pantothenate stores.	upregulation decreases parasite load	As/Pf, As/Py	[44]
retinoid and acid binding glycoprotein (RFABG)	apolipophorin precursor, MQ lipid transport. Source of lipids for developing PL oocysts	parasite load decreases in KDM	Ag/Pb	[45]
trehalose transporter (TreT)	trehalose transport from fat body to haemolymph in MQ. Source of glucose for PL development.	parasite load decreases in KDM	Ag/Pf	[18]
		upregulated in INFM	Ag/Pf	[36]

**Table 3.** Role of non-immune physiological proteins previously reported as being putatively involved in mosquito fecundity. Natural mosquito/Plasmodium combinations are in bold. MQ = mosquito, PL = Plasmodium, Ag = Anopheles gambiae, Ac = Anopheles coluzzi, As = Anopheles stephensi, Aa= Aedes aegypti, Pf = Plasmodium falciparum (human), Pb = Plasmodium berguei (rodent), Py = Plasmodium yoelii (rodent), Pg = Plasmodium gallinaceum (avian), INFM = experimentally infected mosquitoes, KDM = knock-down mosquitoes.

molecule	function	experimental results	MQ/PL combination	references
20-Hydroxyecdysone (20E)	reproductive hormone, involved in MQ egg production. Stimulates Vg synthesis.	downregulated expression in INFM	Aa/Pg	[39]
		downregulation decreases parasite load	Ag/Pf	[40]
allatoicase (ALLC)	converts excess uric acid (antioxidant) into urea in MQ. Contributes to redox homeostasis in MQ. A reduction in uric acid may be correlated to a reduction of fecundity in MQ.	upregulated expression in INFM	Ag/Pb	[46]
branched chain amino acid transferase (BCAT)	involved in egg production in MQ	upregulation decreases parasite load	Ac/Pf	[47]
nutrient sensing pathway (TOR)	source of lipids for energy and membrane neogenesis in MQ. Regulates cellular metabolism. Conversion of blood meal into eggs.	downregulated expression in INFM	Ag/Pf	[36]
urate oxidase (UO)	converts excess uric acid (antioxidant) into urea in MQ. contributes to redox homeostasis in MQ. A reduction in uric acid may be correlated to a reduction of fecundity in MQ.	upregulated expression in INFM	Ag/Pb	[46]
vitellogenin (Vg)	precursor of egg storage protein (vitellin) in MQ	accumulation in haemolymph of INFM	As/Py	[48]
		downregulated expression in INFM	Ag/Py	[49]
		up/downregulated* expression in INFM	Aa/Pg	[39]
vitellogenic cathepsin B (VCB)	digests vitellin granules in eggs, rendering amino acids available for the MQ embryo	up/downregulated* expression in INF	Aa/Pg	[39]

**Table 4.** Role of non-immune physiological proteins previously reported as being putatively involved in *Plasmodium* salivary gland survival and onward transmission. Natural mosquito/Plasmodium combinations are in bold. MQ = mosquito, PL = *Plasmodium*, Ag = *Anopheles gambiae*, As = *Anopheles stephensi*, Aa= Aedes aegypti, Pf = *Plasmodium falciparum* (human), Pb = *Plasmodium berguei* (rodent), Pg = *Plasmodium gallinaceum* (avian), INFM = experimentally infected mosquitoes, KDM = knock-down mosquitoes.

molecule	function	experimental results	MQ/PL combination	references
agaphelin	antihaemostatic, facilitates MQ blood feeding.	upregulated expression in INFM	Ag/Pf	[50]
anophelin	antihaemostatic, facilitates MQ blood feeding.	upregulated expression in INFM	As/Pb	[51]
apyrase (APY)	anti-hemostatic (blood feeding). Low apyrase has been associated to multiple feeding attempts. When ingested with blood meal prevents blood coagulation in MQ midgut.	activity lowered in INFM	Ae/Pg	[52]
		downregulated expression in INFM	Ag/Pb	[53]
		activity lowered in INFM	Ag/Pb	[54]
		supplementation increases parasite load	Ag/Pb	[55]
circumsporozoite-protein binding protein (CSPBP)	MQ salivary gland protein. Putative ligand for salivary gland invasion by PL sporozoites	parasite load decreases in KDM	Ag/Pb	[56]
D7 salivary proteins	salivary gland protein re-ingested by the MQ during the blood meal. Facilitates PL colonization of the midgut (unknown mechanism). Antihemostatic properties.	parasite load decreases in KDM	Ae/Pg	[28]
saglin	ligand for Plasmodium entry into salivary glands Component of MQ saliva, re-ingested by the MQ during the blood meal. Facilitates PL colonization of the midgut.	downregulation decreases sporozoite load	Ag/Pb, Ag/Pf	[57]
		parasite load decreases in KDM	Ac/Pb, Ac/Pf	[58]
SAMSP1	MQ salivary gland protein. Facilitates sporozoite movement and invasion of host hepatocytes	parasite load decreases in KNDM	Ag/Pb	[59]
SGS1	MQ salivary gland protein. Putative ligand for salivary gland invasion by PL sporozoites	parasite load decreases in KDM	Aa/Pg	[60]
TRIO	MQ salivary gland protein. Facilitates sporozoite movement and invasion of host hepatocytes.	upregulated expression in INFM	Ag/Pf	[61]
		sporozoites from KDM colonize the liver less effectively	Ag/Pb	[62]

**Table 5.** Role of non-immune physiological proteins previously reported as being putatively involved in mosquito neuronal function and behaviour. Natural mosquito/Plasmodium combinations are in bold. MQ = mosquito, PL = *Plasmodium*, Ag = *Anopheles gambiae*, Pf = *Plasmodium falciparum* (human), INFM = experimentally infected mosquitoes.

molecule	function	experimental results	MQ/PL combination	references
acetylcholine esterase (Ace)	general neuronal function	upregulated expression in INFM	Ag/Pf	[36]
acetylcholine receptors (AChr)	general neuronal function	upregulated expression in INFM	Ag/Pf	[36]
gamma aminobutyric acid (GABA-A, B)	general neuronal function	upregulated expression in INFM	Ag/Pf	[36]
gustatory receptors (GRs)	gustatory reception. modulates sensitivity to sugary food sources	upregulated expression in INFM	Ag/Pf	[36]
		up/downregulated expression in INFM**	Ag/Pf	[63]
ionotropic receptors (IRs)	olfactory responses to host semiochemicals. host preference and seeking behaviour.	upregulated expression in INFM	Ag/Pf	[36]
		downregulated expression in INFM	Ag/Pf	[63]
odorant binding and chemosensory proteins (OBPs, CSPs)	solubility and binding of odorant molecules	upregulated expression in INFM	Ag/Pf	[36]
		up/downregulated expression in INFM**	Ag/Pf	[63]
odorant receptors (ORs)	odour reception	upregulated expression in INFM	Ag/Pf	[36]
		downregulated expression in INFM*	Ag/Pf	[63]
pickpocket channel	gustatory reception	upregulated expression in INFM	Ag/Pf	[36]
transient receptor potential channels (TRPCs and TRPMs)	gustatory sensitivity	up/downregulated expression in INFM	Ag/Pf	[36]
transient receptor potential channels (TRP-Waterwitch)	moisture detection	downregulated expression in INFM	Ag/Pf	[36]

## 2. Material and methods

### 2.1. Experimental design

The aim of the experiment was to understand how avian malaria infections influence expression patterns of non-immune genes of known relevance to *Plasmodium* development in mosquitoes at four different key stages of *P. relictum* development within the mosquito [64]: 30 min after the blood meal ingestion (30 min) (gametocyte activation and formation of gametes), 8 days post-infection (8 dpi) (peak of oocyst production), 12 dpi (peak sporozoite production) and 22 dpi (end stages of the infection). The full protocol and immune-gene expression data were published in a recent study [8].

Briefly, six canaries, 3 uninfected, 3 infected with *P. relictum* (cytochrome-b lineage pSGS1) were used for the experiments. Ten days later, at the peak of the acute infection stage in blood, the three infected (parasitaemias calculated as % of infected red blood cells: 3.55%, 3.05% and 2.85%) and three control birds were placed individually in an experimental cage with 150 7-day-old female mosquitoes, which had been reared using standard laboratory protocols [65]. Cages were visited 30  min later to take out the bird and any mosquitoes that were not fully gorged. At this point, 10 fully gorged resting mosquitoes were randomly sampled from each of the cages. Although mosquitos could have fed from the bird at any time between 1 and 30  min, the samples were pooled for the analyses as a sampling point (henceforth, ‘30 mpi’). Samples were homogenized with 500  μl of TRIzol LS, and frozen at −80°C for subsequent RNA extraction (one pool of 10 mosquitoes per cage). The rest of the mosquitoes were left in their cages with a source of sugar solution (10%) at our standard insectary conditions (25−27°C, 70% RH). Cages were also supplied with a water container to allow egg laying. On day 8 after the blood meal, 10 mosquitoes were randomly taken from each of the three cages (‘8 dpi’ sample), homogenized with 500  μl of RNAlater and frozen at −80°C. The procedure was repeated on days 12 (‘12 dpi’ sample) and 22 (‘22 dpi’ sample). TRIzol was used for the blood-engorged mosquitoes because bird blood, with its nucleated red blood cells, clogs the filters of the RNA extraction spin columns if they are not first treated in a TRIzol step (see below). To verify the success of the infection, at 8 and 12 dpi, a further sample of 10 mosquitoes per cage was taken and immediately dissected to quantify *Plasmodium* oocysts in the mosquito gut. All the mosquitoes analysed harboured parasites.

### 2.2. RNA extraction

All analyses were carried out using pools of 10 mosquitoes (one pool per time point per bird). For the 30 mpi samples, the total volume of the buffer was adjusted to 750  μl; the sample, containing mosquitoes and buffer, was subsequently homogenized using a TissueLyser (Qiagen) equipped with a 5 mm stainless steel bead. The TissueLyser was run for two cycles of 3 min at 30  Hz. Phase separation was done according to the TRIzol LS manufacturer’s protocol; the resulting aqueous phase was mixed with one volume of 70% ethanol and placed in a RNeasy Mini spin column. RNA from the 8, 12 and 22 dpi samples was extracted by first transferring the mosquitoes to a new tube together with 600  μl of buffer RLT and a 5 mm stainless steel bead and then homogenized using a TissueLyser. The TissueLyser was run for two cycles of 3 min at 30  Hz. Afterwards RNA was extracted using RNeasy Mini spin columns following the manufacturer’s protocol.

The concentration of all RNA samples was measured on a Nanodrop 2000/2000c (Thermo Fisher Scientific). mRNA from each time point was sequenced using an Illumina HiSeq platform at an average of 85 million reads per library (Novogene). We obtained paired-end reads 150 bp in length.

### 2.3. Data processing

We generated a list of 270 non-immune genes, or gene orthologs, that have been identified, with different degrees of confidence, in the literature as being crucial for *Plasmodium* development within the mosquito (see electronic supplementary material, table S1). These genes are involved in crucial physiological pathways within the mosquito, and include structural and digestive proteins, hormones and enzyme involved in the mosquito’s energetic metabolism. At each time point, the expression levels of these genes were compared between infected and control mosquitoes.

Sequence data quality testing was performed using FastQC (version 0.11.8) [66]. Low-quality reads were filtered or trimmed with Trimmomatic (version 0.27) [67]. The resulting files were aligned with Star (version 2.7.9 a) [68] by using the *C. quinquefasciatus* genome as reference [69]. Finally, read count per gene was performed using featureCounts (version 2.0.1.1) [70].

### 2.4. Statistical analyses

All the statistical analyses were carried out with the free statistical software R (R Core Team, 2020) and the free integrated development environment Rstudio (Rstudio Team, 2020). The package DESeq2 (version 1.16.1) [71] was used to estimate the variance–mean dependence in count data from high-throughput sequencing assays and to test for differential expression based on a model using the negative binomial distribution. When testing for significant differences in expression, and to avoid problems arising from sequencing depth, gene length or RNA composition, the count data were first normalized in DESeq2 [72].

To compare gene expression levels between infected and control mosquitoes, we used a differential expression analysis based on the negative binomial (a.k.a. Gamma–Poisson) distribution though the function DESeq in the DESeq2 package [72].

## 3. Results and discussion

In this study, we present a comprehensive transcriptomic analysis of *Plasmodium*-infected mosquitoes, with our findings systematically categorized into five distinct sections based on the potential roles these genes may play in *Plasmodium* fitness within the mosquito vector. The first section delves into genes associated with midgut invasion, providing insights into how *Plasmodium* overcomes the initial barriers to establish infection within the mosquito. The second section focuses on energetic metabolism, highlighting how the parasite utilizes mosquito energetic pathways to support its own energetically demanding development and proliferation. The third section examines genes involved in mosquito life history traits, uncovering how infection influences, in particular, mosquito fecundity. The rationale behind this is that, for horizontally transmitted parasites such as *Plasmodium*, mosquito fecundity is irrelevant to their fitness. Therefore, diverting resources away from egg production towards their own survival and transmission can provide them with an evolutionary advantage, as has been shown in many castrating parasite species [73]. The fourth section explores genes related to salivary gland invasion and onward transmission, elucidating the mechanisms by which *Plasmodium* prepares for transmission to a new host. Finally, the fifth section addresses mosquito biting behaviour, offering a deeper understanding of how genetic changes may enhance the likelihood of successful transmission to humans.

### 3.1. Midgut invasion

Although population reduction occurs at several steps of *Plasmodium’s* life cycle, one of the most drastic bottlenecks takes place between the ingestion of the gametocytes by the mosquito and the production of oocysts on midgut. A study by Gouagna *et al.*, for instance, estimated that in *P. falciparum*, on average only 0.5% of the gametocytes ingested made it to the oocyst stage [74]. During the first few hours of the infection, the gametocytes need to transform into gametes within the midgut. The search for a mysterious ‘gametocyte activating factor’, responsible for the exflagellation of male gametocytes to produce eight flagellated male gametes, was completed when xanthurenic acid (XA), a byproduct of the mosquito’s kynurenine pathway which metabolizes tryptophan to generate eye-colour pigments (ommochromes), was first identified [75–77].

**Figure 1.**
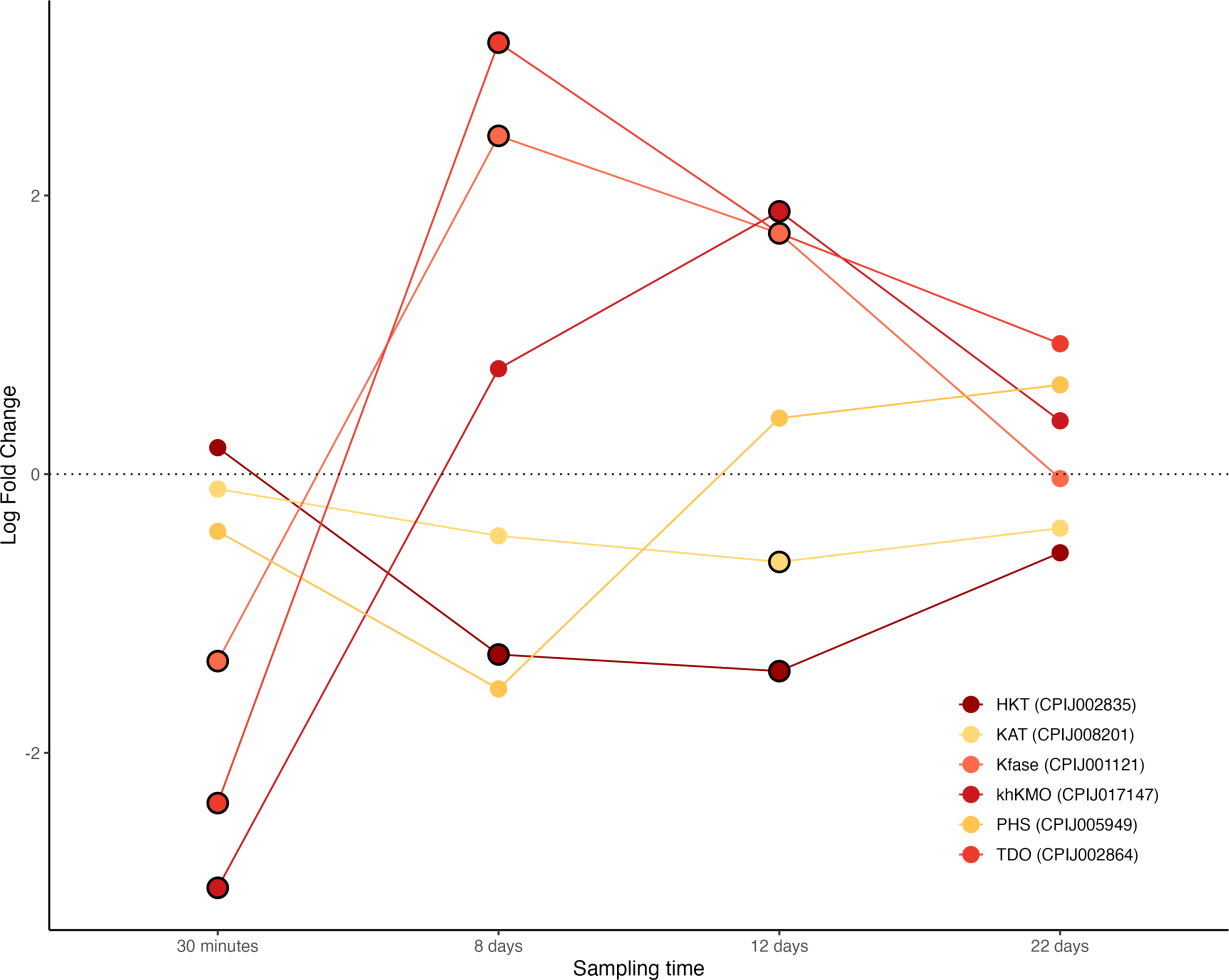
Differential expression pattern of enzymes involved in the kynurenine pathway: tryptophan−2,3-dioxygenase (TDO), kynurenine formamidase (KFase), kynurenine 3-mono-oxygenase (khKMO), 3-hydroxykynurenine transaminase (HKT), kynurenine aminotransferase (KAT) and phenoxazinone synthetase (PHS). Differential expression is represented as log-fold change values. Values above and below the dotted line indicate, respectively, increased and decreased expression in infected mosquitoes with respect to non-infected ones. The black circle around each dot indicates that the difference is statistically significant (adjusted *p* < 0.05).

In our transcriptome data, we were able to quantify all 6 enzymes of the kynurenine pathway (figure 1 and electronic supplementary material, figure S1). The three upstream enzymes that convert tryptophan into the ommochrome precursor 3-hydroxykynurenyne (3HK): tryptophan dioxygenase (TDO), kynurenine formamidase (KFase) and kynurenine mono-oxygenase (KMO) showed a striking pattern of transcript abundance, with significantly lower mRNA levels in the first few minutes after the infection, and significantly higher levels on days 8−12 post infection, during the peak oocyst and sporozoite formation. The fourth enzyme, hydroxykynurenine transaminase (HKT) responsible for transforming 3HK into XA was however, marginally, albeit significantly, lower mRNA levels in infected mosquitoes on days 8−12. Of the two enzymes that shunt substrates away from the pathway, PHS did not exhibit any significant differences between infected and uninfected mosquitoes, whereas KAT showed marginally (albeit statistically significantly) lower mRNA levels on day 12 (figure 1; figure S2).

While the exact timing of *P. relictum* exflagellation is not precisely known, based on data from other *Plasmodium* species, it is reasonable to assume that exflagellation occurs within the first few minutes following gametocyte ingestion. If this is the case, then the lower mRNA levels of the XA biosynthesis enzymes at 30-min post-infection indeed unexpected. One possible explanation is that XA required to trigger exflagellation may be produced or stored in the mosquito prior to blood feeding and thus may not depend on immediate transcriptional activation after infection. Further work quantifying XA metabolite levels is needed to disentangle transcriptional regulation from functional XA availability during early infection.

The decreased expression of HKT in infected mosquitoes contrasts with previous results obtained in *Anopheles stephensi*, a human malaria vector, infected with the rodent malaria parasite *P. berghei* in which this gene showed increased mRNA abundance 8 days after ingestion of infected blood [22] (table 1). This contrasts with patterns observed in human–rodent malaria systems, where similar enzymatic steps in the kynurenine pathway tend to show concordant regulation. Recent work carried out in this system has uncovered a new role for the kynurenine pathway that does not involve the production of XA as a putative gametocyte activating factor [13]. Knocking down HKT, which transforms 3-hydroxykynurenyne (3HK) into XA (electronic supplementary material, figure S1), significantly increased the number of parasite oocysts in the midgut compared to the controls. The resulting accumulation of 3HK, which has a pro-oxidant activity, impaired the structure of the peritrophic matrix (PM), facilitating its traversal by the ookinetes and the formation of oocysts [13]. Further investigation is necessary to determine whether the overexpression of TDO, KFase and KMO, along with the decreased mRNA levels of HKT observed in our experiment, results in an accumulation of 3HK and a resulting increase in oocyst load in infected *Cx. pipiens* mosquitoes. While this pattern could hypothetically indicate a bottleneck in XA production as part of a mosquito counter-response, we consider such an interpretation premature without functional validation, given the complexity of metabolic regulation.

The fusion of the gametes leads to a mobile ookinete that needs to traverse the different layers of the midgut to form an oocyst. There is abundant, albeit controversial, evidence that proteolytic enzymes such as trypsins, chymotrypsins and carboxypeptidases, secreted by the mosquito to digest the blood meal, play a crucial role in the successful formation of oocysts. Baton and Randford-Cartwright suggested that digestive enzymes are largely responsible for the large destruction of ookinetes they observed within the midgut lumen [78]. While they did not directly measure specific enzymes, their discussion references earlier studies showing that the peak activity of trypsin and chymotrypsin occurs earlier in *An. albimanus* than in more susceptible species such as *An. stephensi* [78]. Other studies, however, established that certain digestive enzymes are, on the contrary, essential for the successful development of *Plasmodium* within the midgut [24,25,79].

**Figure 2.**
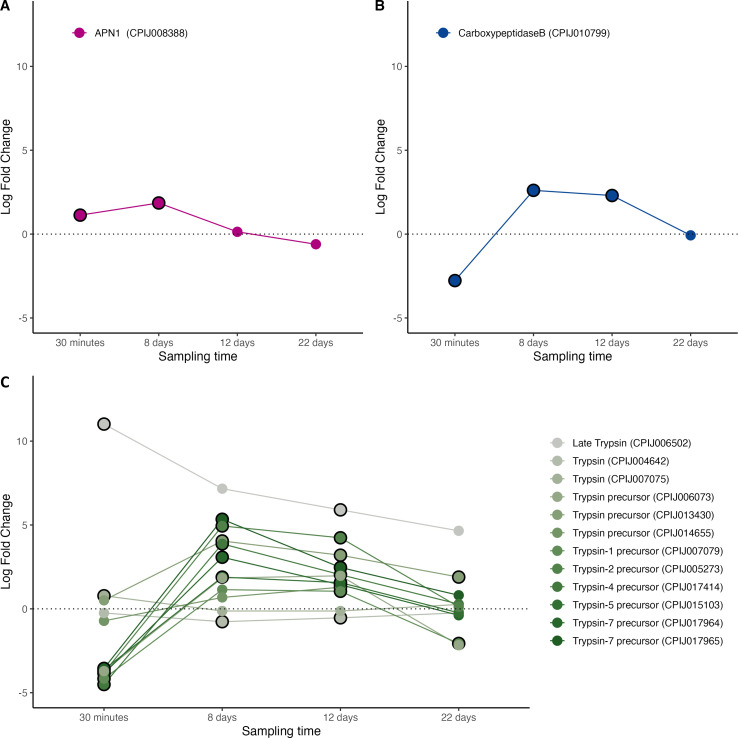
Differential expression pattern of digestive enzymes. (A) Aminopeptidase−1, (B) carboxypeptidase-B, and (C) trypsin andtrypsin precursors (see legend), Differential expression is represented as log-fold change values. Values above and below the dotted line indicate, respectively, increased and decreased expression in infected mosquitoes with respect to non-infected ones. The black circle around each dot indicates that the difference is statistically significant (adjusted *p* < 0.05).

In our dataset, several digestive enzymes showed significantly higher mRNA levels in infected mosquitoes (figure 2A–C, figure S3). These included aminopeptidase-N, a digestive enzyme that also serves as a ligand for *P. falciparum* in the midgut [80] (figure 2A), carboxypeptidase-B, which favours *P. falciparum* and *P. berghei* oocyst development in the midgut possibly by providing lysine and arginine for the parasite [24,79], and several trypsin and trypsin precursors (figure 2C). Trypsin is essential for the activation of the *Plasmodium* chitinase, the enzyme that allows the parasite to traverse the peritrophic membrane [14,15,35]. Carboxypeptidase-A, which has been previously shown to favour oocyst development in the midgut through unknown mechanisms [24], was not found in our transcript database. Our results therefore fully agree with previous studies showing an increased expression of these digestive enzymes in infected mosquitoes (table 1) which may be suggestive of the parasite’s ability to manipulate these enzymes to its own advantage.

**Figure 3.**
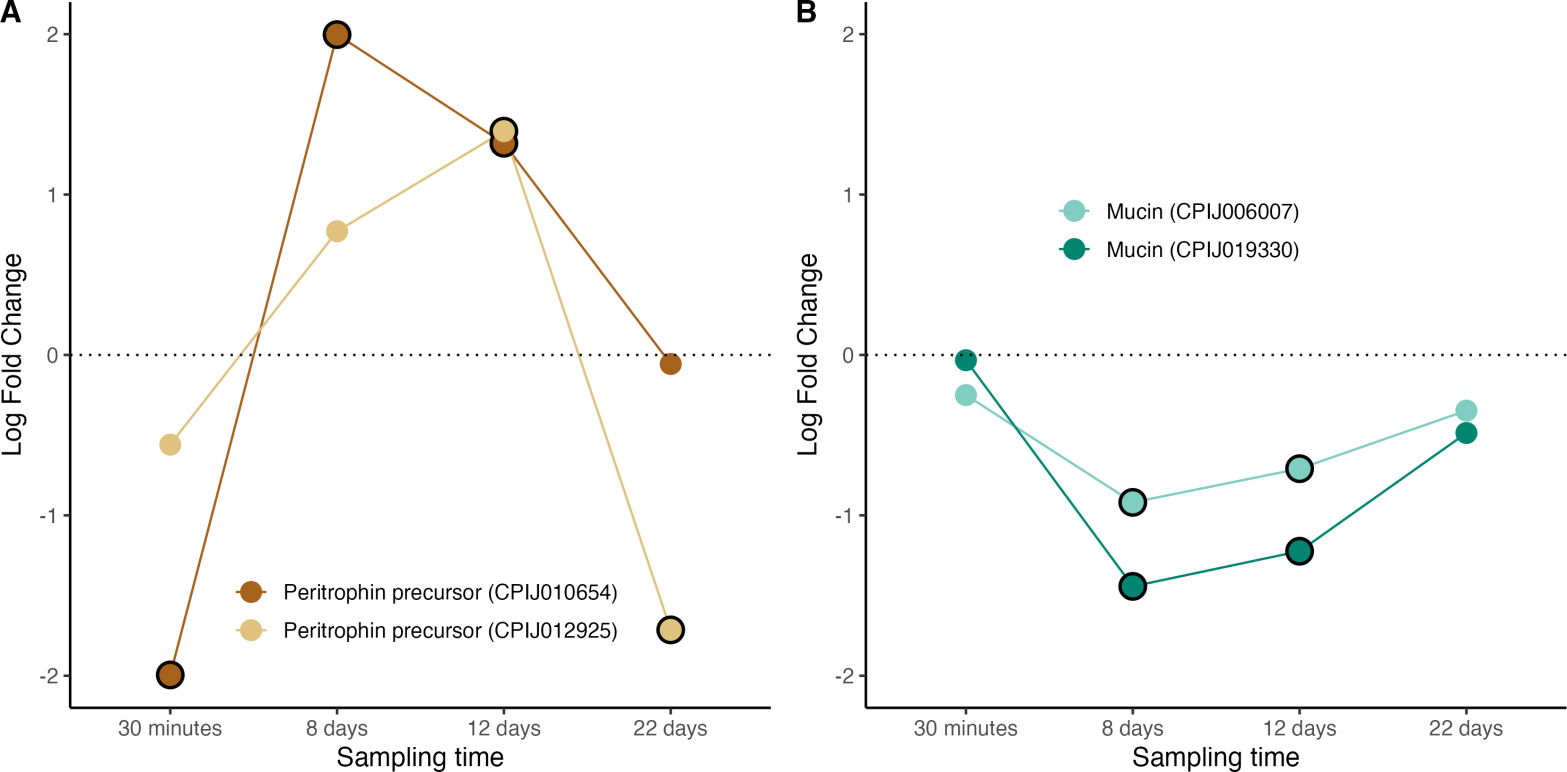
Differential expression pattern of peritrophic matrix (PM) and glycocalyx protein components. (A) Peritrophin precursors and (B) mucins. Differential expression is represented as log-fold change values. Values above and below the dotted line indicate, respectively, increased and decreased expression in infected mosquitoes with respect to non-infected ones. The black circle around each dot indicates that the difference is statistically significant (adjusted *p* < 0.05).

Two extracellular structures have been proposed to provide protection to the midgut epithelium in mosquitoes: the peritrophic matrix (PM) and the glycocalyx [81]. These two structures represent a barrier for the invasion of the midgut epithelial cells by *Plasmodium*. The peritrophic matrix is an extracellular sac secreted by the gut epithelial cells, composed of chitin, proteins and proteoglycans, which completely surrounds the ingested blood meal. Peritrophins are one of the key proteins responsible for the integrity of the PM [23]. None of the peritrophin genes identified from *Anopheles* mosquitoes in VectorBase were found in our transcripts. We did find two genes labelled as PM precursors, one of which (CPIJ010654) was significantly down regulated in infected mosquitoes during the early stages of the infection (30 min, figure 3A, figure S4). Both precursors where significantly upregulated 8 and 12 days post infection. The absence of annotated Anopheles peritrophin orthologs in our dataset may reflect genuine differences in midgut invasion strategies between Culex and Anopheles, or may simply be due to the comparatively lower annotation quality of the Culex pipiens genome.

Past the peritrophic matrix, the luminal side of epithelial cells is covered with the carbohydrate-rich glycocalyx. Mucin is one of the key glycoprotein constituents of the glycocalyx. Previous work has established that knocking down the MUC1 gene results in a reduction of *P. gallinaceum* oocysts by over 50% [31], and that silencing genes responsible for maintaining the integrity of the mucin barrier, results in a significant reduction of *P. falciparum* and *P. berghei* oocysts in mosquitoes [30,32]. These results suggest that a defective mucin barrier increases the permeability of the midgut to immune effectors, consequently leading to a significant reduction in *Plasmodium* oocysts in the midgut [30]. Interestingly, two mucin genes were identified in our database, both of which were found to be highly significantly down-regulated in *Plasmodium*-infected mosquitoes (figure 3B, figure S4), suggesting that *Plasmodium* may be able to modulate the expression of these genes in order to maximize its survival in the midgut.

### 3.2. Energetic metabolism

There is abundant experimental evidence that *Plasmodium* requires extensive carbohydrate and lipid resources in order to fulfil its energetic needs within the mosquito (reviewed in [82]). The most resource-hungry stage of *Plasmodium* within mosquitoes are the oocysts, which constitute veritable DNA-replicating machines, with each oocyst producing thousands of sporozoites. There is increasing evidence that the parasite is heavily dependent on a panel mosquito lipid [16,17] and sugar [18,19] transport proteins as well as enzymatic [20] and hormonal pathways [21] to provide the developing oocysts with these crucial resources.

**Figure 4.**
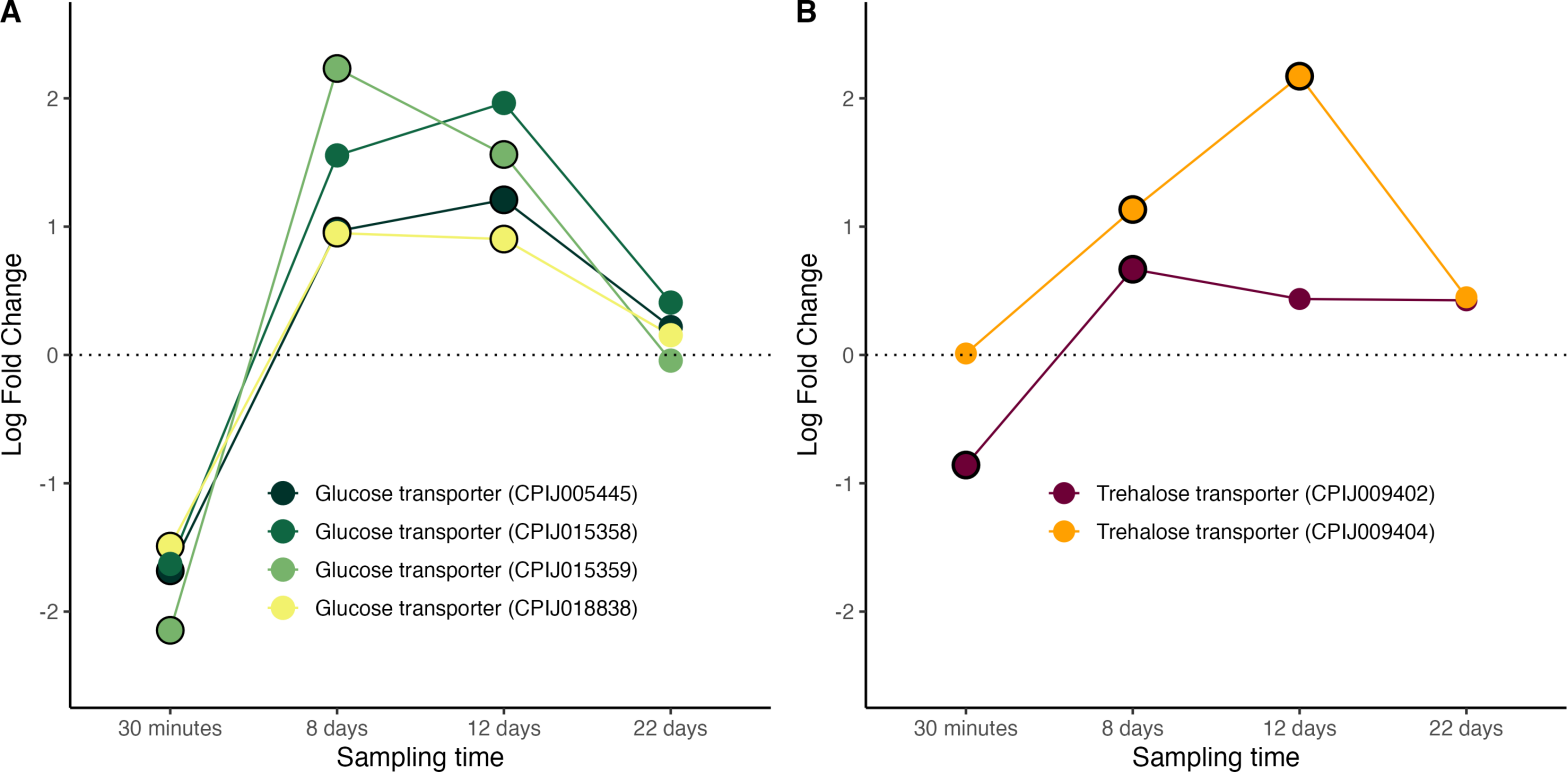
Differential expression pattern of genes involved in sugar transport. (A) Glucose transporter genes and (B) trehalose transporter genes. Differential expression is represented as log-fold change values. Values above and below the dotted line indicate, respectively, increased and decreased expression in infected mosquitoes with respect to non-infected ones. The black circle around each dot indicates that the difference is statistically significant (adjusted *p* < 0.05).

Several important genes involved in sugar and lipid metabolism were found to be significantly upregulated in infected mosquitoes during the oocyst-formation stage of the *Plasmodium* life cycle. Trehalose transporters are responsible for the transportation of trehalose from the fat body to the haemolymph while glucose transporters mediate the movement of sugars into the cells [19]. Our transcriptome recovered 4 different glucose and 2 different trehalose transporter genes, most of which were significantly over-expressed at 8−12 days post infection, coinciding with the window of oocyst maturation (figure 4A,B, figure S5). These results, which agree with previously published studies using other mosquito-*Plasmodium* combinations (see table 2), could be interpreted in two non-exclusive ways, highlighting the inherent difficulty of interpreting transcriptomic studies as evidence (or lack thereof) of parasite manipulation. On the one hand, it could be a *Plasmodium*-driven strategy whereby the parasite manipulates the expression of these genes to fulfil its energetic means which are known to be consequential [19,83–85]. On the other hand, this could be a mosquito-driven strategy at fulfilling the glucose needs associated to mounting an immune response to the parasite [86].

**Figure 5.**
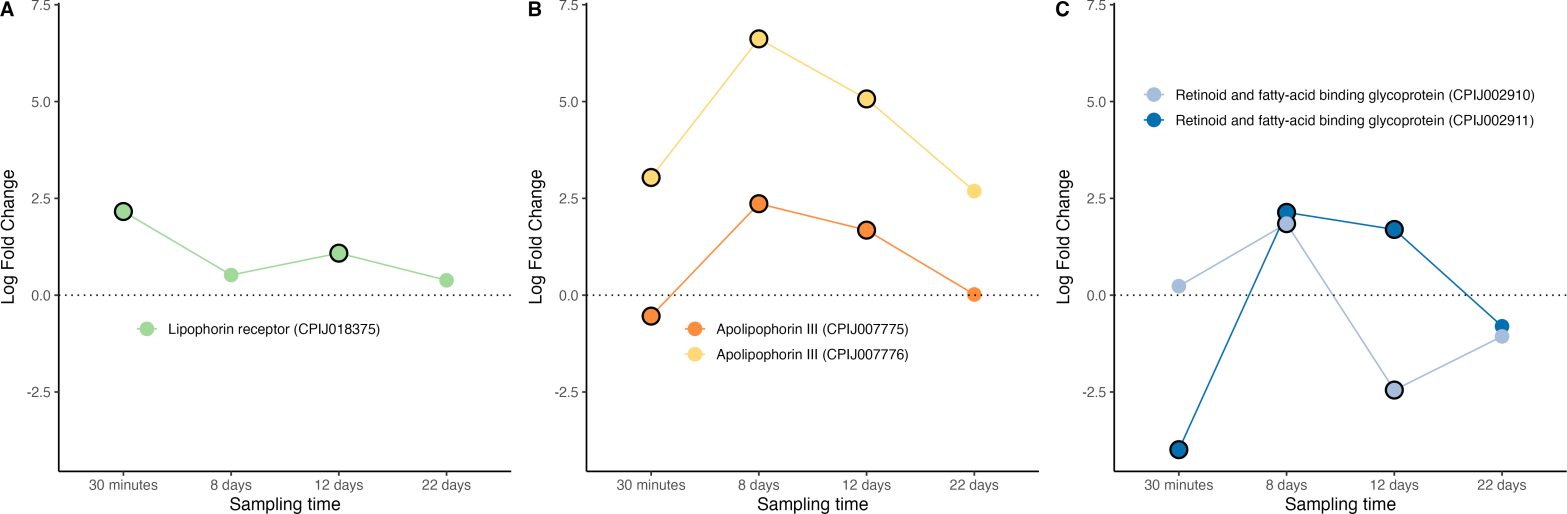
Differential expression pattern of genes involved in lipid transport and metabolism. (A) lipophorin receptor, (B) apolipophorin III, (C) retinoid and fatty-acid binding glycoprotein (RFABG). Differential expression is represented as log-fold change values. Values above and below the dotted line indicate, respectively, increased and decreased expression in infected mosquitoes with respect to non-infected ones. The black circle around each dot indicates that the difference is statistically significant (adjusted *p* < 0.05).

The main lipid carriers in mosquitoes are lipoproteins called lipophorins [38]. The lipophorin particle consists of two apolipoproteins: apolipoprotein-I and apolipoprotein-II. There is evidence that apoLp-I and apoLp-II are the product of the same gene and that the two proteins arise from a posttranslational proteolytic processing event [38]. A third apolipophorin, apoLp-III can be found as a lipid-free hemolymph protein that associates with lipophorin during hormone-induced lipid mobilization. Besides its participation in lipid transport, in mosquitoes apoLp-III has also been reported to mediate midgut epithelial defence responses that limit a *P. berghei* infection [87]. We recovered transcripts from one lipophorin receptor (figure 5A), two apoLp-III (figure 5B), and one lipophorin precursor (RFABG: retinoid and fatty-acid binding glycoprotein, figure 5C) all of which were significantly upregulated during the peak oocyst/sporozoite production phase of the infection (days 8−12) (figure S6). These results largely agree with findings in other systems (table 2).

**Figure 6.**
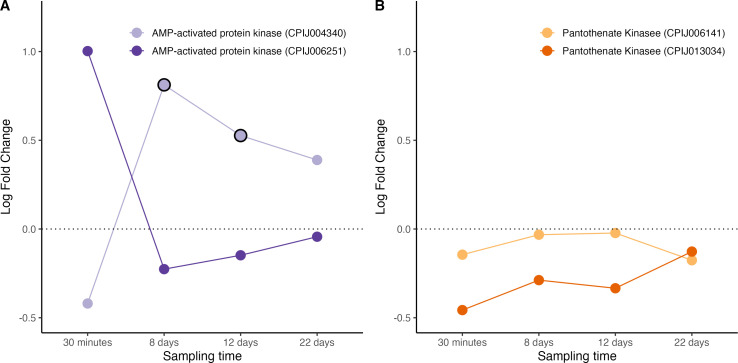
Differential expression pattern of AMP protein kinase (A) and pantothenate kinase (B) genes. Differential expression is represented as log-fold change values. Values above and below the dotted line indicate, respectively, increased and decreased expression in infected mosquitoes with respect to non-infected ones. The black circle around each dot indicates that the difference is statistically significant (adjusted *p* < 0.05).

AMP-activated protein kinase (AMPK) is a key metabolic energy sensor that regulates total energy stores in many organisms, including mosquitoes [37]. When activated, AMPK inhibits anabolic pathways such as glycogen, protein and fatty acid synthesis and activates catabolic processes that synthesize ATP. AMPK activation also inhibits acetyl CoA carboxylase, crucial in fatty acid synthesis, resulting in decreased levels of cellular lipids [37]. We recovered transcripts from two AMPK genes, one of which showed significant overexpression in infected mosquitoes as compared to uninfected ones on days 8−12 (figure 6A, figure S7). Given AMPK’s known role in suppressing reproduction and regulating energy homoeostasis, one possible interpretation is that increased AMPK activity reflects a parasite-mediated manipulation of host metabolism. This could benefit the parasite by redirecting resources away from reproduction (a form of parasite-induced castration) or by stabilizing host energy metabolism during the metabolically demanding phase of parasite development. Alternatively, elevated AMPK expression may represent a component of the mosquito’s physiological response to infection, aiming to maintain energy balance or limit damage associated with infection. These results are however contrary to previous work showing diminished AMPK transcripts in the salivary glands of *An. gambiae* mosquitoes infected with *P. falciparum* [36]. The difference in tissue specificity may partly account for the contrasting patterns of AMPK expression observed. Other enzymes implicated in the CoA cycle also showed significant modifications in infected vs uninfected mosquitoes. CoA is synthesized from a precursor called pantothenate via the enzyme pantothenate kinase (PanK). *Plasmodium* parasites cannot synthesize pantothenate, and must therefore uptake it from their mosquito hosts. Previous work has shown that an upregulation of PanK starves *P. falciparum* and *P. yoelii* of pantothenate, resulting in significant decreases in parasite numbers [44]. We saw no up or down regulation in either of the two PanK genes we identified in the transcripts of *Cx. pipiens* infected mosquitoes (figure 6B, figure S7).

### 3.3. Fecundity and nutrient recycling

Many parasite species have evolved strategies to reduce the fecundity of their hosts, redirecting resources originally intended for egg production towards their own survival and proliferation [73,88]. Insect eggs are rich in yolk proteins and lipids, which are essential for embryonic development. Since *Plasmodium* transmits only horizontally, mosquito fecundity is of no direct consequence for parasite’s fitness, and studies on mosquito-*Plasmodium* interactions have consistently shown that the parasite frequently reduces mosquito fecundity [89,90]. This has prompted suggestions that, in line with other parasite species, *Plasmodium* may have been selected to either reduce egg production or increase egg resorption in its mosquito host. By redirecting these key resources towards its own metabolic needs and/or towards extending the mosquito lifespan, the parasite would considerably enhance its transmission opportunities. Previous work carried out in our laboratory has shown that a *P. relictum* infection is associated with a significant decrease in fecundity and an increase in mosquito longevity, providing support for this parasite’s ability to reallocate host resources to enhance its own transmission potential [90].

Mosquito ovaries work as conveyor belts where the primary egg follicles are in a pre-vitellogenic state until a blood meal is taken, which triggers a hormonal cascade that initiates the production of yolk protein precursors in the fat body [91]. Once the primary egg follicles are laid, a second burst of reproductive hormones makes the secondary follicles enter a pre-vitellogenic stage where their development is arrested until a second blood meal is taken. At least four key metabolic regulators are involved in egg production in mosquitoes: the target of rapamycin (TOR) nutritional signalling pathway, along with the hormones ecdysone, and juvenile hormone (JH), and insulin-like peptides [92]). TOR is an evolutionary conserved gene that links elevated hemolymph amino acid levels derived from the blood meal to the expression of yolk protein precursors in the fat body. JH stimulates growth and development of the egg follicles and renders them competent to respond to ecdysone [91]. Fat body cells take up ecdysone, convert it to 20-hydroxyecdysone (20E) and use it to activate transcription of vitellogenin genes, the genes encoding the major egg-yolk proteins [93].

**Figure 7.**
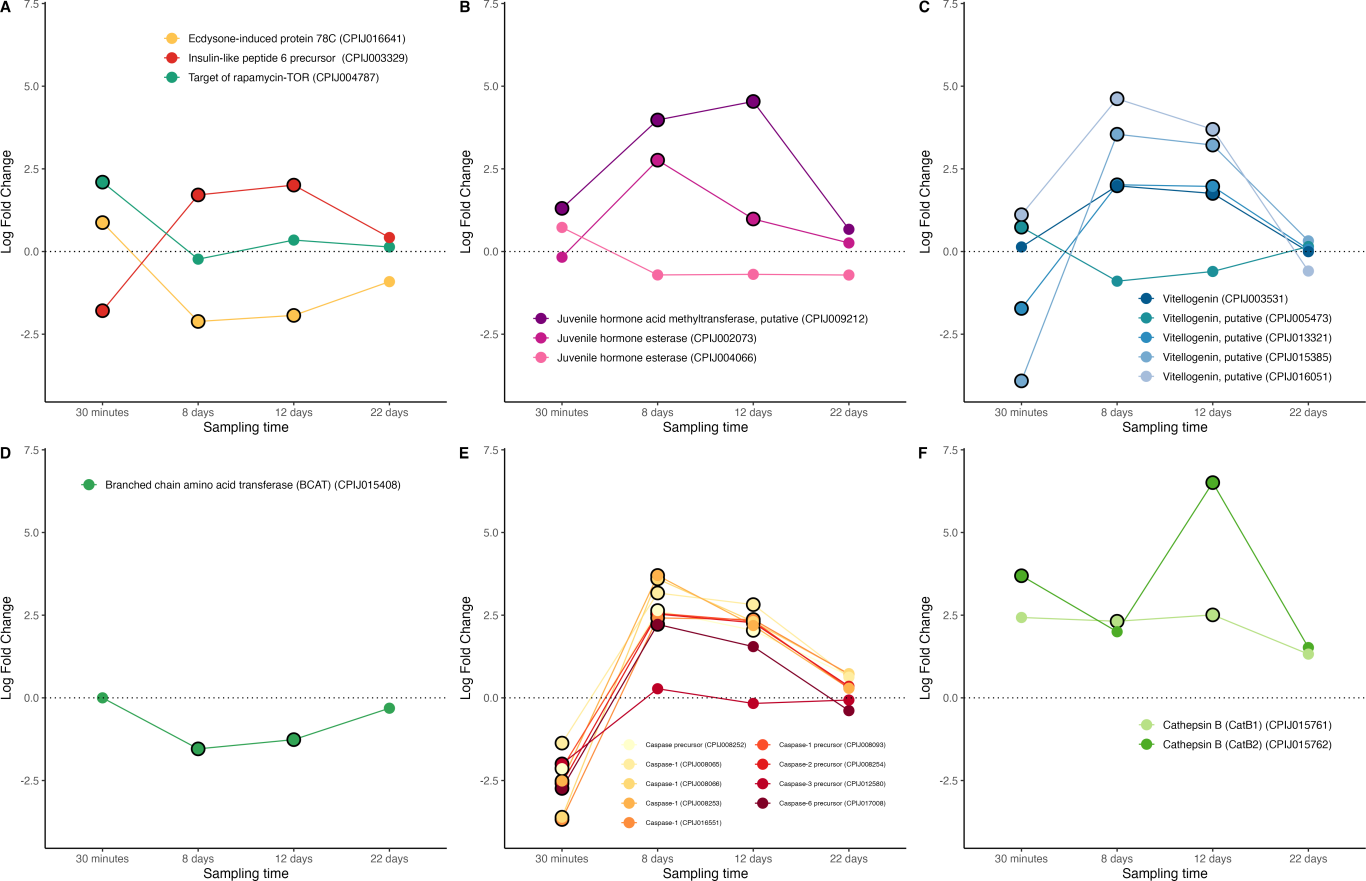
Differential expression pattern of genes involved in egg production and resorption. (A) Ecdysone, insulin-like peptide and TOR genes (B) juvenile hormone, (C) vitellogenin, (D) BCAT, (E) caspases, (F) cathepsin B. Differential expression is represented as log-fold change values. Values above and below the dotted line indicate, respectively, increased and decreased expression in infected mosquitoes relative to non-infected ones. The black circle around each dot indicates that the difference is statistically significant (adjusted *p* < 0.05).

In our experiment, mosquitoes laid their first batch of eggs 3−4 days after the blood meal, so mosquitoes sampled on days 8−22 could not have had any fully mature eggs, only eggs in a pre-vitellogenic state (although this was not verified). Despite this, we observed marked differences between infected and uninfected mosquitoes in metabolic pathways involved in egg production (figure S8). We observed a higher TOR mRNA abundance in infected mosquitoes immediately after the blood meal, but no significant differences thereafter (figure 7A). Infected mosquitoes had, however, significantly higher levels of two JH transcripts than their uninfected counterparts particularly on days 8−12 post blood meal (figure 7B). Although we did not detect any ecdysone transcripts, immediately after the blood meal-infected mosquitoes exhibited significantly higher mRNA levels of the ecdysone-response gene E78, a protein that plays a crucial role in egg production in *Drosophila* [94], though has not yet been described in mosquitoes. The mRNA level of this protein decreases drastically relative to that of uninfected mosquitoes between days 8−10 post blood meal (figure 7C). The levels of a precursor of insulin-like peptide (ILPs), which work synergistically with 20E to activate transcription of vitellogenin genes showed an opposite pattern. mRNA abundance was over twofold lower in infected than in uninfected mosquitoes immediately after the bloodmeal and were twofold higher on days 8−12 (figure 7D). We also retrieved transcripts from five vitellogenin or putative vitellogenin genes, four of which showed higher abundance on *Plasmodium*-infected mosquitoes on days 8−12 (figure 7E). Recently, the enzyme branched-chain aminoacid transferase (BCAT) has been shown to play a crucial role in egg production in mosquitoes [47]. Knocking-down its upstream regulator (miR−276) increases BCAT production, which in turn enhances fecundity and decreases *P. falciparum* burden [47]. Interestingly, our results show a drastic down regulation of BCAT in infected, relative to uninfected, mosquitoes on days 8−12 after the blood meal (figure 7F).

Egg resorption is a process that has evolved in many insect species to redirect nutritional resources stored in eggs to other physiological processes of the organism [95]. In mosquitoes placed under stressful conditions, both pre-vitellogenic and vitellogenic follicles may be resorbed [95,96]. Follicle atresia is one of the main factors contributing to malaria-induced fecundity reduction in mosquitoes [89,97]. Two proteolytic enzymes seem to play a central role in egg resorption in mosquitoes: caspases, a family of cysteine proteases that play essential roles in the regulation and execution of apoptosis [89,95] and cathepsin B, a cysteine peptidase that has been shown to be involved in vitellin degradation in mosquitoes [98,99]. We recovered transcripts from nine different caspases or caspase precursors and two cathepsins, B1 and B2 (figure 7E,F). They all show a pattern of mRNA abundance that is consistent with a *Plasmodium*-induced trigger of follicular atresia: infected mosquitoes had up to a fourfold increase in caspase mRNA levels between days 8−12 post blood meal and up to a sevenfold increase in cathepsin B1 mRNA levels compared to uninfected mosquitoes. These results should, however, be interpreted with caution, considering that caspases, in particular, have been shown to play significant roles in other apoptotic processes associated with *Plasmodium* infection. Most notably, ookinete invasion of the midgut epithelium triggers a caspase-mediated apoptosis of the midgut cells [100] which is why caspases are often considered to be part of the mosquito immune system [101]. Previous work carried out on *P. falciparum* has shown that the parasite is able to evade this arm of the immune system by inhibiting the activity of caspases in the midgut of *An. gambiae* mosquitoes [101,102]. Our findings of significantly higher caspase mRNA levels at days 8−12 post infection are in stark contrast to these previous results.

### 3.4. Salivary gland invasion and transmission

**Figure 8.**
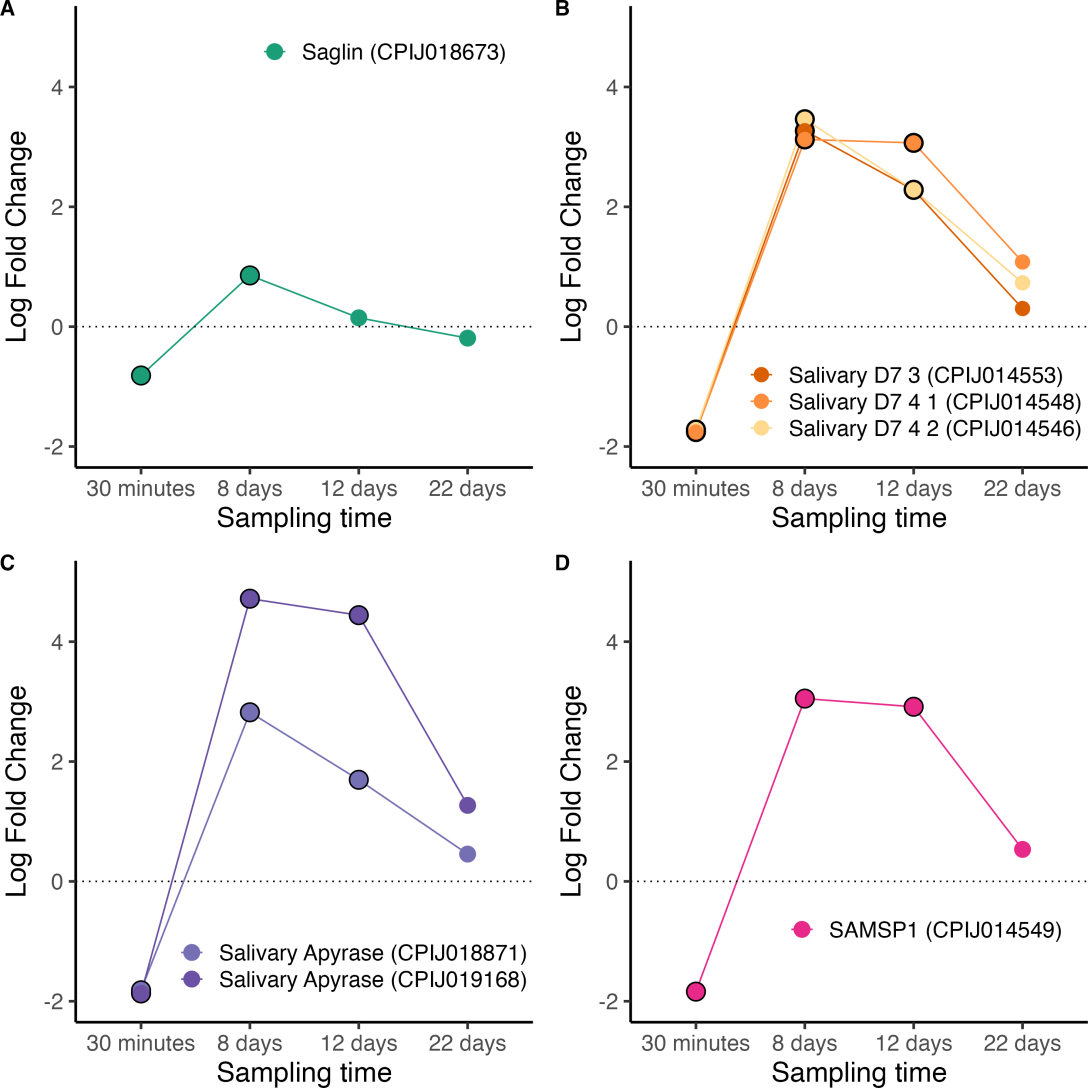
Differential expression pattern of salivary gland proteins. (A) Saglin, (B) Salivary D7 proteins, (C) apyrase and (D) SAMSP1. Differential expression is represented as log-fold change values. Values above and below the dotted line indicate, respectively, increased and decreased expression in infected mosquitoes relative to non-infected ones. The black circle around each dot indicates that the difference is statistically significant (adjusted *p* < 0.05).

The salivary glands play a crucial role in *Plasmodium* development because they host the transmissible stages of the parasites (the sporozoites). Sporozoite invasion of the mosquito salivary glands have been shown to require the interaction between *Plasmodium’*s thrombospondin‐related anonymous protein (TRAP) and a salivary gland receptor in mosquitoes called saglin. Saglin knock-downs resulted in severely decreased sporozoite loads in the salivary glands of mosquitoes infected with *P. falciparum* and *P. berghei* [57]. More recently, however, Klug *et al.* [58] have discovered a different role for saglin in the life cycle of these two parasite species. They found that mosquitoes with a knocked-down version of the gene had substantially fewer midgut (oocyst) stages of the parasite than their wild-type counterparts. Their results strongly suggest that saglin is secreted in the saliva and re-ingested during the blood meal, facilitating the colonization of the mosquito midgut through unknown mechanisms [103]. Our results on *P. relictum*-infected *Cx. pipiens* mosquitoes show a marginal (albeit statistically significant) decrease in saglin mRNA levels at the beginning of the infection (30 min) followed by an increase at the peak of oocyst formation in the midgut on day 8 (figure 8A, figure S9). However, no significant changes in saglin transcripts were detected during the sporozoite stage of the parasite cycle (days 12−22) as would have been expected if the parasite were able to manipulate this salivary gland protein to its advantage.

Previous work has shown that a *Plasmodium* infection has profound effects in several other excreted salivary proteins of mosquitoes [104]. Our transcripts recovered three of these excreted proteins: D7, apyrase and sporozoite-associated mosquito salivary protein (SAMSP1). D7 proteins are among the most abundant components of the mosquito saliva. There are two subfamilies of D7 proteins in mosquitoes: the long-forms (7–30 kDa), and the short-forms (15–20 kDa). Although the role of D7 proteins in *Culex* mosquitoes has not yet been entirely elucidated, recent work has shown that two D7 long forms, L1 and L2, have a dual role. On the one hand, they have antihemostatic properties that prevent clotting at the site of the mosquito bite, with knock-down mosquitoes having longer probing times than their wild-type counterparts [105]. On the other hand, as with saglin, these salivary gland proteins are re-ingested with the blood meal facilitating the oocyst invasion of the midgut through unknown mechanisms [28]. We obtained transcripts for three D7 proteins, all of which showed significantly lower mRNA levels in infected mosquitoes at 30 min post-blood meal ingestion, followed by significantly higher thereafter (figure 8B, figure S9).

The first mosquito salivary gland protein to be identified as having a key role in *Plasmodium* transmission was apyrase [52]. Apyrase has been shown to have anti-hemostatic properties, and there are consistent results showing that *P. berghei* and *P. gallinaceum* lower apyrase activity [52,54] and expression levels [53] resulting in prolonged probing times [52]. More recent, though at the time of writing unpublished, results suggest that mosquitoes ingest a substantial amount of apyrase during blood feeding which reduces coagulation in the blood meal by enhancing fibrin degradation and inhibiting platelet aggregation [55]. Supplementation of *Plasmodium* infected blood with apyrase significantly enhanced *P. berghei* infection in the mosquito midgut [55]. We obtained transcripts from two salivary apyrase genes, both of which showed significantly higher mRNA levels in infected versus uninfected mosquitoes immediately after the feeding event (30 min) and significantly higher levels at 8 and 12 days post-infection (figure 8C, figure S9). Although the role of SAMPSP1 in mosquitoes has not yet been elucidated, this protein has been recently shown to aid the gliding motility and cell traversal activity of *P. berghei* sporozoites [59]. In our experiment SAMPSP1 followed the same pattern of mRNA abundance as D7 and apyrase in infected relative to uninfected mosquitoes: significantly lower levels at 30 min followed by significantly higher levels during peak sporozoite (day 8) and peak sporozoite (day 12) production (figure 8D, figure S9).

### 3.5. Mosquito behaviour

The purported manipulation of mosquito behaviour by *Plasmodium* stands out as one of the most extensively documented instances of parasite manipulation [106]. The mechanistic basis for this manipulation remains, however, largely unexplored. Infected and uninfected mosquitoes have shown differential responses to compounds in host odour using electroantennography [107], and transcriptomic studies carried out in heads or antennae of *An. gambiae* mosquitoes infected with *P. falciparum* sporozoites have shown complex shifts in transcription patterns of key odorant and gustatory genes [36,63].

**Figure 9.**
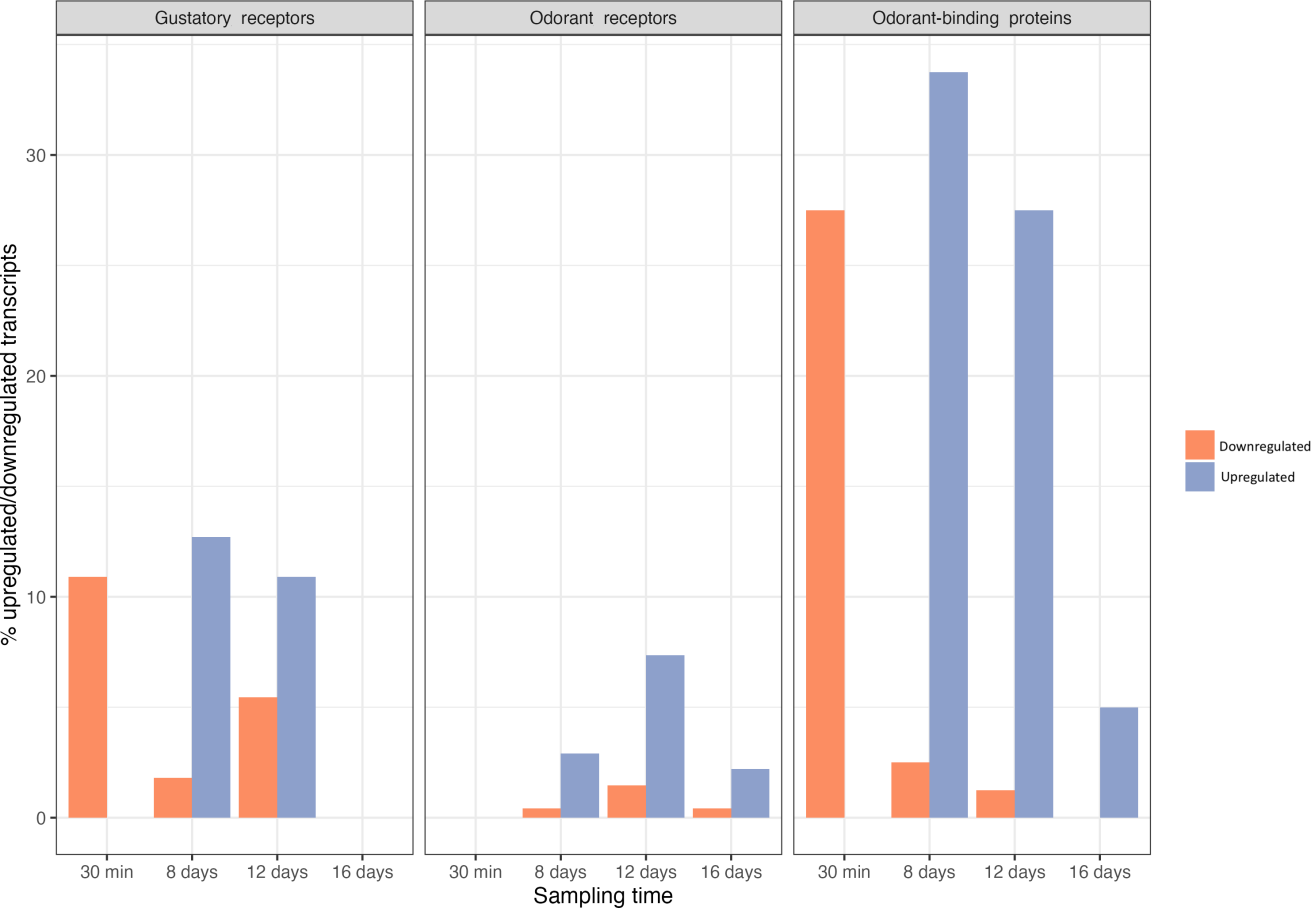
Percentage of gustatory receptors, odorant receptors and odorant-binding protein transcripts that are significantly down (orange) or up (blue) regulated.

We detected genes coding for 91 odorant receptors (ORs), 62 odorant-binding (OBPs) and chemosensory proteins (CSPs) and 28 gustatory receptors (GRs, figure 9). The largest differences between infected and uninfected mosquitoes were observed in OBPs, with 27.5% of genes significantly downregulated shortly after the blood meal (30 min) and 27.5–33.7% significantly upregulated during peak oocyst and sporozoite production (days 8−12, figure 9). A similar, though less pronounced, pattern was found in GRs, where 10.9% of genes were downregulated immediately after the blood meal (30 min) and upregulated on days 8 and 12. The smallest changes were seen in ORs, where on days 8−12, the differences between upregulated (2.9–7.4%) and downregulated (0.4–1.5%) genes were less notable. Our results indicate a significant shift towards greater odour and gustatory sensitivity of the mosquito during the transmissible stages of the infection. These results broadly agree with recent transcriptomic studies carried out in heads of *An. gambiae* mosquitoes infected with *P. falciparum* sporozoites [36] and are congruent with studies showing significant shifts in mosquito behaviour during a *Plasmodium* infection. Mosquitoes infected with *Plasmodium* have demonstrated increased attraction to vertebrate hosts, greater persistence in biting, and a higher frequency of feeding when they are carrying the infective sporozoite stage of the parasite [108–110] (but see [111]). These behavioural modifications enhance transmission opportunities for the parasite and have therefore been widely interpreted as being the result of parasite manipulation. [9,110] demonstrated that these shifts in behaviour were not exclusive to *Plasmodium* infections. Mosquitoes infected with *Escherichia coli* exhibited similar shifts in feeding preferences, leading the researchers to conclude that this shift, possibly mediated by insulin-like peptides (ILPs) in the midgut, was not due to parasitic manipulation but rather the mosquito’s immune response to infection [9]. The parasite, they posited, may have evolved to profit from this shift in mosquito physiology.

## 4. Conclusion

*P. relictum* infection significantly impacts a multitude of physiological pathways in *Cx. pipiens*, in ways that can potentially boost its development and maximize its transmission rates. We observed a significant upregulation of key genes involved in sugar and lipid metabolism, during the crucial oocyst-formation stage, at a time when the parasite critically needs these resources to build thousands of sporozoites, each with its own membrane, haploid genome, cytoplasm and organelles. We also observed significant alterations in key metabolic pathways essential for egg production and resorption, indicating a disrupted reproductive system. These changes provide insights into the mechanisms behind the reduction in fecundity associated with malaria infection in mosquitoes [90,112,113]. This reduction in reproductive output, which in *P. relictum*-infected mosquitoes has been shown to be as high as 40%, has been shown to be associated with a significant increase in mosquito longevity, a crucial determinant of parasite transmission [90]. Integrative studies combining molecular data (e.g. transcriptomics, proteomics, metabolomics) with trait-level measurements (e.g. egg counts, follicle staging and oocyte resorption) in *Plasmodium*-infected mosquitoes are long overdue and would greatly enhance our understanding of parasite-associated changes in mosquito fecundity. Additionally, transcripts for several salivary gland proteins showed significant variations in expression, consistent with previous findings that proteins like apyrase and D7 play crucial roles in enhancing *Plasmodium* transmission. Significant shifts in mRNA abundance related to odorant and gustatory receptors in mosquitoes infected with *Plasmodium*, particularly during the transmissible stages of the infection, also align with previous research indicating increased infected mosquito attraction to vertebrate hosts [114].

The difficulty lies in establishing whether these shifts are the result of parasite manipulation or simply the mosquito’s response to the infection, which the parasite may serendipitously exploit to enhance its fitness. From an evolutionary perspective, identifying whether these modifications are driven by the parasite or the vector is a crucial step towards understanding the selective pressures acting on this infectious system. Short of identifying the precise mechanism through which *Plasmodium* may be manipulating its host, something that to our knowledge has not yet been accomplished, one option would be to follow Cator *et al*.’s approach [9,110] and test whether infections with non-vector-borne pathogens yield the same results. An interesting follow-up to this study would thus be to determine which, if any, of the observed transcriptional modifications observed in *Cx pipiens* are absent in mosquitoes infected with *E coli*.

These findings raise important questions about the evolutionary strategies employed by different *Plasmodium* species. The contrast between the generalist nature of *P. relictum* and the host/vector specialization seen in *P. falciparum* suggests that distinct mechanisms, such as host castration, immune modulation or behavioural manipulation, may underlie their interaction with mosquito vectors. Exploring these differences could reveal key trade-offs between transmission efficiency and parasite specialization. Furthermore, the discrepancies observed across studies regarding mosquito responses to infection highlight the need for future research aimed at disentangling the ecological and evolutionary drivers of such variation. These open questions offer promising starting points for understanding how parasite strategies shape transmission dynamics under natural conditions.

Due to the ongoing climate change, avian malaria is projected to become more frequent. Rising temperatures and changing weather patterns are expanding mosquito habitats, increasing the range of avian malaria and affecting previously unexposed endemic bird species. This shift disrupts ecosystems and threatens biodiversity. Addressing this issue requires a multi-faceted approach, including monitoring vector populations, and developing strategies to block the development of the parasite within the mosquito. The concept of mosquito population replacement, driven by advancements in gene-driven technologies, is emerging as a promising malaria control strategy. This strategy relies on the construction of transgenic mosquitoes that are resistant to *Plasmodium* through a variety of different blocking mechanisms to prevent the parasite from evolving resistance. Although the most widely studied examples of this approach utilize the mosquito’s immune responses, the use of mosquito-derived pathways that facilitate parasite infection and transmission is a promising avenue [115]. Our study uncovers for the first time the specific physiologic pathways that *P. relictum* exploits to thrive within its mosquito vector, providing critical insights for developing targeted interventions to disrupt these pathways and inhibit parasite development.

## Data Availability

Sequences have been uploaded to the Sequence Read Archive (SRA) at NCBI under accession no.: PRJNA848963. Supplementary material is available online [116].

## References

[1] Rivero A, Gandon S. 2018 Evolutionary ecology of Avian malaria: past to present. Trends Parasitol. **34**, 712–726. (10.1016/j.pt.2018.06.002) 29937414

[2] Atkinson CT, Dusek RJ, Woods KL, Iko WM. 2000 Pathogenicity of avian malaria in experimentally-infected Hawaii Amakihi. J. Wildl. Dis. **36**, 197–204. (10.7589/0090-3558-36.2.197) 10813599

[3] Woodworth BL *et al*. 2005 Host population persistence in the face of introduced vector-borne diseases: Hawaii amakihi and avian malaria. Proc. Natl Acad. Sci. USA **102**, 1531–1536. (10.1073/pnas.0409454102) 15668377 PMC547860

[4] Bensch S, Hellgren O, Pérez‐Tris J. 2009 MalAvi: a public database of malaria parasites and related haemosporidians in avian hosts based on mitochondrial cytochrome b lineages. Mol. Ecol. Resour. **9**, 1353–1358. 21564906 10.1111/j.1755-0998.2009.02692.x

[5] Loiseau C, Harrigan RJ, Bichet C, Julliard R, Garnier S, Lendvai ÁZ, Chastel O, Sorci G. 2013 Predictions of avian Plasmodium expansion under climate change. Sci. Rep. **3**, 1126. (10.1038/srep01126) 23350033 PMC3553554

[6] Ielmini MR, Sankaran KV. 2000 100 of the world’s worst invasive alien species: a selection from the global invasive species database. Auckland: Invasive Species Specialist Group (IUCN). See https://portals.iucn.org/library/sites/library/files/documents/2000-126.pdf.

[7] Clayton AM, Dong YM, Dimopoulos G. 2014 The Anopheles innate immune system in the defense against malaria infection. J. Inn. Immun. **6**, 169–181. (10.1159/000353602) PMC3939431 23988482

[8] García‐Longoria L, Ahrén D, Berthomieu A, Kalbskopf V, Rivero A, Hellgren O. 2023 Immune gene expression in the mosquito vector Culex quinquefasciatus during an avian malaria infection. Mol. Ecol. **32**, 904–919. (10.1111/mec.16799) 36448733 PMC10108303

[9] Cator LJ, Pietri JE, Murdock CC, Ohm JR, Lewis EE, Read AF, Luckhart S, Thomas MB. 2015 Immune response and insulin signalling alter mosquito feeding behaviour to enhance malaria transmission potential. Sci. Rep. **5**, 11947. (10.1038/srep11947) 26153094 PMC4495552

[10] Sinden RE. 2015 The cell biology of malaria infection of mosquito: advances and opportunities. Cell. Microbiol. **17**, 451–466. (10.1111/cmi.12413) 25557077 PMC4409862

[11] Dash M, Sachdeva S, Bansal A, Sinha A. 2022 Gametogenesis in Plasmodium: delving deeper to connect the dots. Front. Cell. Infect. Microbiol. **12**, 877907. (10.3389/fcimb.2022.877907) 35782151 PMC9241518

[12] Guttery DS, Zeeshan M, Holder AA, Tromer EC, Tewari R. 2023 Meiosis in Plasmodium: how does it work? Trends Parasitol. **39**, 812–821. (10.1016/j.pt.2023.07.002) 37541799

[13] Feng Y, Peng Y, Song X, Wen H, An Y, Tang H, Wang J. 2022 Anopheline mosquitoes are protected against parasite infection by tryptophan catabolism in gut microbiota. Nat. Microbiol. **7**, 707–715. (10.1038/s41564-022-01099-8) 35437328

[14] Huber M, Cabib E, Miller LH. 1991 Malaria parasite chitinase and penetration of the mosquito peritrophic membrane. Proc. Natl Acad. Sci. USA **88**, 2807–2810. (10.1073/pnas.88.7.2807) 2011589 PMC51328

[15] Shahabuddin M, Toyoshima T, Aikawa M, Kaslow DC. 1993 Transmission-blocking activity of a chitinase inhibitor and activation of malarial parasite chitinase by mosquito protease. Proc. Natl Acad. Sci. USA **90**, 4266–4270. (10.1073/pnas.90.9.4266) 8483942 PMC46487

[16] Atella GC, Silva-Neto MAC, Golodne DM, Arefin S, Shahabuddin M, Alberto M, Silva-Neto C. 2006 Anopheles gambiae lipophorin: characterization and role in lipid transport to developing oocyte. Insect Biochem. Mol. Biol. **36**, 375–386. (10.1016/j.ibmb.2006.01.019) 16651184

[17] Costa G *et al*. 2018 Non-competitive resource exploitation within mosquito shapes within-host malaria infectivity and virulence. Nat. Commun. **9**, 3474. (10.1038/s41467-018-05893-z) 30150763 PMC6110728

[18] Liu K, Dong Y, Huang Y, Rasgon JL, Agre P. 2013 Impact of trehalose transporter knockdown on Anopheles gambiae stress adaptation and susceptibility to Plasmodium falciparum infection. Proc. Natl Acad. Sci. USA **110**, 17504–17509. (10.1073/pnas.1316709110) 24101462 PMC3808592

[19] Wang M, Wang J. 2020 Glucose transporter GLUT1 influences Plasmodium berghei infection in Anopheles stephensi. Parasit. Vectors **13**, 1–11. (10.1186/s13071-020-04155-6) 32503601 PMC7275331

[20] Thakre N *et al*. 2022 Manipulation of pantothenate kinase in Anopheles stephensi suppresses pantothenate levels with minimal impacts on mosquito fitness. Ins. Biochem. Mol. Biol. **149**, 103834. (10.1016/j.ibmb.2022.103834) PMC9595603 36087890

[21] Nyasembe VO, Hamerly T, López-Gutiérrez B, Leyte-Vidal AM, Coatsworth H, Dinglasan RR. 2023 Adipokinetic hormone signaling in the malaria vector Anopheles gambiae facilitates Plasmodium falciparum sporogony. Commun. Biol. **6**, 14. (10.1038/s42003-023-04518-6) 36782045 PMC9924834

[22] Xu X *et al*. 2005 Transcriptome analysis of Anopheles stephensi-Plasmodium berghei interactions. Mol. Biochem. Parasitol. **142**, 76–87. (10.1016/j.molbiopara.2005.02.013) 15907562

[23] Dinglasan RR, Devenport M, Florens L, Jacobs-Lorena M, Johnson M JR, Donnelly-Doman C, Carucci M, Yates D JR. 2009 The Anopheles gambiae adult midgut peritrophic matrix proteome. Insect. Biochem. Mol. Biol. **39**, 125–134. (10.1016/j.ibmb.2008.10.010) 19038338 PMC2684889

[24] VenkatRao V, Kumar SK, Sridevi P, Muley VY, Chaitanya RK. 2017 Cloning, characterization and transmission blocking potential of midgut carboxypeptidase A in Anopheles stephensi. Acta Trop. **168**, 21–28. (10.1016/j.actatropica.2016.12.035) 28087198

[25] Lavazec C, Boudin C, Lacroix R, Bonnet S, Diop A, Thiberge S, Boisson B, Tahar R, Bourgouin C. 2007 Carboxypeptidases B of Anopheles gambiae as targets for a Plasmodium falciparum transmission-blocking vaccine. Infect. Immun. **75**, 1635–1642. (10.1128/IAI.00864-06) 17283100 PMC1865713

[26] Raz A, Dinparast Djadid N, Zakeri S. 2013 Molecular characterization of the carboxypeptidase B1 of Anopheles stephensi and its evaluation as a target for transmission-blocking vaccines. Infect. Immun. **81**, 2206–2216. 23569111 10.1128/IAI.01331-12 PMC3676044

[27] Marie A, Holzmuller P, Tchioffo MT, Rossignol M, Demettre E, Seveno M, Cornelie S. 2014 Anopheles gambiae salivary protein expression modulated by wild Plasmodium falciparum infection: highlighting of new antigenic peptides as candidates of An. gambiae bites. Parasit. Vectors **7**, 599. 25526764 10.1186/s13071-014-0599-y PMC4287575

[28] Martin-Martin I *et al*. 2023 Aedes aegypti D7 long salivary proteins modulate blood feeding and parasite infection. mBio **14**, e0228923. (10.1128/mbio.02289-23) 37909749 PMC10746281

[29] Zhang G, Niu G, Franca CM, Dong Y, Wang X, Butler NS, Dimopoulos G, Li J. 2015 Anopheles midgut FREP1 mediates Plasmodium invasion. J. Biol. Chem. **290**, 16490–16501. 25991725 10.1074/jbc.M114.623165 PMC4505404

[30] Kumar S, Molina-Cruz A, Gupta L, Rodrigues J, Barillas-Mury C. 2010 A peroxidase/dual oxidase system modulates midgut epithelial immunity in Anopheles gambiae. Science **327**, 1644–1648. (10.1126/science.1184008) 20223948 PMC3510679

[31] Berois M, Severson DW, Romero‐severson J. 2012 RNAi knock‐downs support roles for the mucin‐like (AeIMUC1) gene and short‐chain dehydrogenase/reductase (SDR) gene in Aedes aegypti susceptibility to Plasmodium gallinaceum. Med. Vet. Entomol. **26**, 112–115. (10.1111/j.1365-2915.2011.00965.x) 21615441 PMC3165091

[32] Kajla M, Kakani P, Choudhury TP, Kumar V, Gupta K, Dhawan R, Gupta L, Kumar S. 2017 Anopheles stephensi heme peroxidase HPX15 suppresses midgut immunity to support Plasmodium development. Front. Immunol. **8**, 249. (10.3389/fimmu.2017.00249) 28352267 PMC5348522

[33] Molina-Cruz A, Canepa GE, Alves e Silva TL, Williams AE, Nagyal S, Yenkoidiok-Douti L, Barillas-Mury C. 2020 Plasmodium falciparum evades immunity of anopheline mosquitoes by interacting with a Pfs47 midgut receptor. Proc. Natl Acad. Sci. USA **117**, 2597–2605. 31969456 10.1073/pnas.1917042117 PMC7007573

[34] Cui Y, Niu G, Li VL, Wang X, Li J. 2020 Analysis of blood-induced Anopheles gambiae midgut proteins and sexual stage Plasmodium falciparum interaction reveals mosquito genes important for malaria transmission. Sci. Rep. **10**, 14316. 32868841 10.1038/s41598-020-71186-5 PMC7459308

[35] Sriwichai P, Rongsiryam Y, Jariyapan N, Sattabongkot J, Apiwathnasorn C, Nacapunchai D, Paskewitz S. 2012 Cloning of a trypsin-like serine protease and rxpression patterns furing Plasmodium falciparum invasion in the mosquito, Anopheles dirus. Arch. Ins. Biochem. Physiol. **80**, 151–165. (10.1002/arch.21034) 22627911

[36] Carr AL, Rinker DC, Dong Y, Dimopoulos G, Zwiebel LJ. 2021 Transcriptome profiles of Anopheles gambiae harboring natural low-level Plasmodium infection reveal adaptive advantages for the mosquito. Sci. Rep. **11**, 22578. (10.1038/s41598-021-01842-x) 34799605 PMC8604914

[37] Oringanje C, Delacruz LR, Han Y, Luckhart S, Riehle MA. 2021 Overexpression of activated AMPK in the Anopheles stephensi midgut impacts mosquito metabolism, reproduction and Plasmodium resistance. Genes **12**, 119. (10.3390/genes12010119) 33478058 PMC7835765

[38] Cheon HM, Shin SW, Bian G, Park JH, Raikhel AS. 2006 Regulation of lipid metabolism genes, lipid carrier protein lipophorin, and its receptor during immune challenge in the mosquito Aedes aegypti. J. Biol. Chem. **281**, 8426–8435. (10.1074/jbc.M510957200) 16449228

[39] Araujo RV, Maciel C, Hartfelder K, Capurro ML. 2011 Effects of Plasmodium gallinaceum on hemolymph physiology of Aedes aegypti during parasite development. J. Insect Physiol. **57**, 265–273. 21112329 10.1016/j.jinsphys.2010.11.016

[40] Werling K, Shaw WR, Itoe MA, Westervelt KA, Marcenac P, Paton DG, Catteruccia F. 2019 Steroid hormone function controls non-competitive Plasmodium development in Anopheles. Cell **177**, 315–325. 30929905 10.1016/j.cell.2019.02.036 PMC6450776

[41] Dhawan R, Gupta K, Kajla M, Kakani P, Choudhury TP, Kumar S, Gupta L. 2017 Apolipophorin-III acts as a positive regulator of Plasmodium development in Anopheles stephensi. Front. Physiol. **8**, 185. 28439240 10.3389/fphys.2017.00185 PMC5383653

[42] Pinheiro-Silva R, Borges L, Coelho LP, Cabezas-Cruz A, Valdés JJ, Do Rosario V, Domingos A. 2015 Gene expression changes in the salivary glands of Anopheles coluzzii elicited by Plasmodium berghei infection. Parasit. Vectors **8**, 485. 26395987 10.1186/s13071-015-1079-8 PMC4580310

[43] Wang M, Wang J. 2020 Glucose transporter GLUT1 influences Plasmodium berghei infection in Anopheles stephensi. Parasit. Vectors **13**, 285. 32503601 10.1186/s13071-020-04155-6 PMC7275331

[44] Simão-Gurge RM, Thakre N, Strickland J, Isoe J, Delacruz LR, Torrevillas BK, Rodriguez AM, Riehle MA, Luckhart S. 2021 Activation of Anopheles stephensi pantothenate kinase and coenzyme a biosynthesis reduces infection with diverse Plasmodium species in the mosquito host. Biomolecules **11**, 807. (10.3390/biom11060807) 34072373 PMC8228300

[45] Vlachou D, Schlegelmilch T, Christophides GK, Kafatos FC. 2005 Functional genomic analysis of midgut epithelial responses in Anopheles during Plasmodium invasion. Curr. Biol. **15**, 1185–1195. 16005290 10.1016/j.cub.2005.06.044

[46] Kwon H, Smith R. 2022 Anopheles gambiae actively metabolizes uric acid following Plasmodium infection to limit malaria parasite survival. Front. Physiol. **12**, 821869. 35140633 10.3389/fphys.2021.821869 PMC8818946

[47] Lampe L, Jentzsch M, Kierszniowska S, Levashina EA. 2019 Metabolic balancing by miR-276 shapes the mosquito reproductive cycle and Plasmodium falciparum development. Nat. Commun. **10**, 5634. (10.1038/s41467-019-13627-y) 31822677 PMC6904670

[48] Jahan N, Hurd H. 1998 Effect of Plasmodium yoelii nigeriensis (Haemosporidia: Plasmodiidae) on Anopheles stephensi (Diptera: Culicidae) vitellogenesis. J. Med. Entomol. **35**, 956–961. 9835686 10.1093/jmedent/35.6.956

[49] Ahmed AM, Maingon R, Romans P, Hurd H. 2001 Effects of malaria infection on vitellogenesis in Anopheles gambiae during two gonotrophic cycles. Insect Mol. Biol. **10**, 347–356. 11520358 10.1046/j.0962-1075.2001.00273.x

[50] Waisberg M, Molina-Cruz A, Mizurini DM, Gera N, Sousa BC, Ma D, Francischetti IM. 2014 Plasmodium falciparum infection induces expression of a mosquito salivary protein (Agaphelin) that targets neutrophil function and inhibits thrombosis without impairing hemostasis. PLoS Pathog. **10**, e1004338. 25211214 10.1371/journal.ppat.1004338 PMC4161438

[51] Dixit R, Sharma A, Mourya DT, Kamaraju R, Patole MS, Shouche YS. 2009 Salivary gland transcriptome analysis during Plasmodium infection in malaria vector Anopheles stephensi. Int. J. Infect. Dis. **13**, 636–646. 19128996 10.1016/j.ijid.2008.07.027

[52] Rossignol PA, Spielman A, Ribeiro JMC. 1984 Increased intradermal probing time in sporozoite-infected mosquitoes. Am. J. Trop. Med. Hyg. **33**, 17–20. (10.4269/ajtmh.1984.33.17) 6696175

[53] Rosinski-Chupin I *et al*. 2007 Serial analysis of gene expression in Plasmodium berghei salivary gland sporozoites. BMC Genom. **8**, 466. (10.1186/1471-2164-8-466) PMC2263065 18093287

[54] Thiévent K, Zilio G, Hauser G, Koella JC. 2019 Malaria load affects the activity of mosquito salivary apyrase. J. Insect Physiol. **116**, 10–16. (10.1016/j.jinsphys.2019.04.003) 30986373

[55] Pala ZR *et al*. 2023 Anopheles salivary apyrase regulates blood meal hemostasis and drives malaria parasite transmission. bioRxiv (10.1101/2023.05.22.541827) PMC11410810 39294191

[56] Wang J, Zhang Y, Zhao YO, Li MW, Zhang L, Dragovic S, Fikrig E. 2013 Anopheles gambiae circumsporozoite protein–binding protein facilitates Plasmodium infection of mosquito salivary glands. J. Infect. Dis. **208**, 1161–1169. 23801601 10.1093/infdis/jit284 PMC3762383

[57] Ghosh AK, Devenport M, Jethwaney D, Kalume DE, Pandey A, Anderson VE, Sultan AA, Kumar N, Jacobs-Lorena M. 2009 Malaria parasite invasion of the mosquito salivary gland requires interaction between the Plasmodium TRAP and the Anopheles saglin proteins. PLoS Pathog. **5**, e1000265. (10.1371/journal.ppat.1000265) 19148273 PMC2613030

[58] Klug D, Gautier A, Calvo E, Marois E, Blandin SA. 2023 The salivary protein saglin facilitates efficient midgut colonization of Anopheles mosquitoes by malaria parasites. PLoS Pathog. **19**, e1010538. (10.1371/journal.ppat.1010538) 36862755 PMC10013899

[59] Chuang YM, Agunbiade TA, Tang XD, Freudzon M, Almeras L, Fikrig E. 2021 The effects of a mosquito salivary protein on sporozoite traversal of host cells. J. Infect. Dis. **224**, 544–553. (10.1093/infdis/jiaa759) 33306099 PMC8328219

[60] Korochkina S, Barreau C, Pradel G, Jeffery E, Li J, Natarajan R, Vernick KD. 2006 A mosquito‐specific protein family includes candidate receptors for malaria sporozoite invasion of salivary glands. Cell. Microbiol. **8**, 163–175. 16367875 10.1111/j.1462-5822.2005.00611.x

[61] Marie A *et al*. 2014 Anopheles gambiae salivary protein expression modulated by wild Plasmodium falciparum infection: highlighting of new antigenic peptides as candidates of An. gambiae bites. Parasit. Vectors **7**, 599. (10.1186/s13071-014-0599-y) 25526764 PMC4287575

[62] Chuang YM, Freudzon M, Yang J, Dong Y, Dimopoulos G, Fikrig E. 2019 Anopheles gambiae lacking AgTRIO inefficiently transmits Plasmodium berghei to mice. Infect. Immun. **87**, 10–1128. 10.1128/IAI.00326-19 PMC6704594 31285253

[63] Hajkazemian M, Hill SR, Mozūraitis R, Ranford-Cartwright L, Emami SN, Ignell R. 2022 Mosquito host-seeking diel rhythm and chemosensory gene expression is affected by age and Plasmodium stages. Sci. Rep. **12**, 18814. (10.1038/s41598-022-23529-7) 36335172 PMC9637142

[64] Sekar V, Rivero A, Pigeault R, Gandon S, Drews A, Ahren D, Hellgren O. 2021 Gene regulation of the avian malaria parasite Plasmodium relictum, during the different stages within the mosquito vector. Genomics **113**, 2327–2337. (10.1016/j.ygeno.2021.05.021) 34023365

[65] Vézilier J, Nicot A, Gandon S, Rivero A. 2010 Insecticide resistance and malaria transmission: infection rate and oocyst burden in Culex pipiens mosquitoes infected with Plasmodium relictum. Malar. J. **9**, 379. (10.1186/1475-2875-9-379) 21194433 PMC3313086

[66] Andrews S. 2010 FastQC: a quality control tool for high throughput sequence data. Babraham, UK: The Babraham Institute.

[67] Bolger AM, Lohse M, Usadel B. 2014 Trimmomatic: a flexible trimmer for Illumina sequence data. Bioinformatics **30**, 2114–2120. (10.1093/bioinformatics/btu170) 24695404 PMC4103590

[68] Dobin A, Davis CA, Schlesinger F, Drenkow J, Zaleski C, Jha S, Batut P, Chaisson M, Gingeras TR. 2013 STAR: ultrafast universal RNA-seq aligner. Bioinformatics **29**, 15–21. (10.1093/bioinformatics/bts635) 23104886 PMC3530905

[69] Arensburger P *et al*. 2010 Sequencing of Culex quinquefasciatus establishes a platform for mosquito comparative genomics. Science **330**, 86–88. (10.1126/science.1191864) 20929810 PMC3740384

[70] Liao Y, Smyth GK, Shi W. 2014 featureCounts: an efficient general purpose program for assigning sequence reads to genomic features. Bioinformatics **30**, 923–930. (10.1093/bioinformatics/btt656) 24227677

[71] Love MI, Huber W, Anders S. 2014 Moderated estimation of fold change and dispersion for RNA-seq data with DESeq2. Genome Biol. **15**, 550. (10.1186/s13059-014-0550-8) 25516281 PMC4302049

[72] Bushel PR, Ferguson SS, Ramaiahgari SC, Paules RS, Auerbach SS. 2020 Comparison of normalization methods for analysis of TempO-Seq targeted RNA sequencing data. Front. Genet. **11**, 594. (10.3389/fgene.2020.00594) 32655620 PMC7325690

[73] Lafferty KD, Kuris AM. 2009 Parasitic castration: the evolution and ecology of body snatchers. Trends Parasitol. **25**, 564–572. (10.1016/j.pt.2009.09.003) 19800291

[74] Gouagna LC *et al*. 1998 The early sporogonic cycle of Plasmodium falciparum in laboratory‐infected Anopheles gambiae : an estimation of parasite efficacy. Trop. Med. Int. Health **3**, 21–28. (10.1046/j.1365-3156.1998.00156.x) 9484964

[75] Billker O, Lindo V, Panico M, Etienne AE, Paxton T, Dell A, Rogers M, Sinden RE, Morris HR. 1998 Identification of xanthurenic acid as the putative inducer of malaria development in the mosquito. Nature **392**, 289–292. (10.1038/32667) 9521324

[76] Jiang Y *et al*. 2020 An intracellular membrane protein GEP1 regulates xanthurenic acid induced gametogenesis of malaria parasites. Nat. Commun. **11**, 1764. (10.1038/s41467-020-15479-3) 32273496 PMC7145802

[77] Bennink S, Pradel G. 2021 Vesicle dynamics during the egress of malaria gametocytes from the red blood cell. Mol. Biochem. Parasitol. **243**, 111372. (10.1016/j.molbiopara.2021.111372) 33961918

[78] Baton LA, Ranford-Cartwright LC. 2012 Ookinete destruction within the mosquito midgut lumen explains Anopheles albimanus refractoriness to Plasmodium falciparum (3D7A) oocyst infection. Int. J. Parasitol. **42**, 249–258. (10.1016/j.ijpara.2011.12.005) 22366731 PMC3401372

[79] Lavazec C, Bourgouin C. 2008 Mosquito-based transmission blocking vaccines for interrupting Plasmodium development. Microbes Infect. **10**, 845–849. (10.1016/j.micinf.2008.05.004) 18656409

[80] Dinglasan RR, Kalume DE, Kanzok SM, Ghosh AK, Muratova O, Pandey A, Jacobs-Lorena M. 2007 Disruption of Plasmodium falciparum development by antibodies against a conserved mosquito midgut antigen. Proc. Natl Acad. Sci. USA **104**, 13461–13466. (10.1073/pnas.0702239104) 17673553 PMC1948931

[81] Shen Z, Dimopoulos G, Kafatos FC, Jacobs-Lorena M. 1999 A cell surface mucin specifically expressed in the midgut of the malaria mosquito Anopheles gambiae. Proc. Natl Acad. Sci. USA **96**, 5610–5615. (10.1073/pnas.96.10.5610) 10318932 PMC21908

[82] Adedeji EO, Ogunlana OO, Fatumo S, Beder T, Ajamma Y, Koenig R, Adebiyi E. 2020 Anopheles metabolic proteins in malaria transmission, prevention and control: a review. Parasit. Vectors **13**, 1–30. (10.1186/s13071-020-04342-5) 32912275 PMC7488410

[83] Rivero A, Ferguson HM, Ferguson H. 2003 The energetic budget of Anopheles stephensi infected by Plasmodium chabaudi: is energy depletion a mechanism for virulence? Proc. R. Soc. B **270**, 1365–1371. (10.1098/rspb.2003.2389) PMC1691381 12965027

[84] Atella GC, Bittencourt-Cunha PR, Nunes RD, Shahabuddin M, Silva-Neto MAC. 2009 The major insect lipoprotein is a lipid source to mosquito stages of malaria parasite. Acta Trop. **109**, 159–162. (10.1016/j.actatropica.2008.10.004) 19013123

[85] Nyasembe VO, Teal PEA, Sawa P, Tumlinson JH, Borgemeister C, Torto B. 2014 Plasmodium falciparum Infection Increases Anopheles gambiae attraction to nectar sources and sugar uptake. Curr. Biol. **24**, 217–221. (10.1016/j.cub.2013.12.022) 24412210 PMC3935215

[86] Dolezal T, Krejcova G, Bajgar A, Nedbalova P, Strasser P. 2019 Molecular regulations of metabolism during immune response in insects. Insect. Biochem. Mol. Biol. **109**, 31–42. (10.1016/j.ibmb.2019.04.005) 30959109

[87] Gupta L *et al*. 2010 Apolipophorin-III mediates antiplasmodial epithelial responses in Anopheles gambiae (G3) mosquitoes. PLoS One **5**, e15410. (10.1371/journal.pone.0015410) 21072214 PMC2970580

[88] Hurd H. 2009 Chapter 4 evolutionary drivers of parasite‐induced changes in insect life‐history traits. In Natural history of host-parasite interactions advances in parasitology (ed. JP Webster), pp. 85–110. Amsterdam, The Netherlands: Academic Press. (10.1016/s0065-308x(08)00604-0) 19289191

[89] Hopwood JA, Ahmed AM, Polwart A, Williams GT, Hurd H. 2001 Malaria-induced apoptosis in mosquito ovaries: a mechanism to control vector egg production. J. Exp. Biol. **204**, 2773–2780. (10.1242/jeb.204.16.2773) 11683433

[90] Vézilier J, Nicot A, Gandon S, Rivero A. 2012 Plasmodium infection decreases fecundity and increases survival of mosquitoes. Proc. R. Soc. B **279**, 4033–4041. (10.1098/rspb.2012.1394) PMC3427586 22859589

[91] Clements AN. 1992 The biology of mosquitoes: development, nutrition and reproduction. London, UK: Chapman & Hall.

[92] Hansen IA, Attardo GM, Rodriguez SD, Drake LL. 2014 Four-way regulation of mosquito yolk protein precursor genes by juvenile hormone-, ecdysone-, nutrient-, and insulin-like peptide signaling pathways. Front. Physiol. **5**, 103. (10.3389/fphys.2014.00103) 24688471 PMC3960487

[93] Dana AN, Hong YS, Kern MK, Hillenmeyer ME, Harker BW, Lobo NF, Hogan JR, Romans P, Collins FH. 2005 Gene expression patterns associated with blood-feeding in the malaria mosquito Anopheles gambiae. BMC Genom. **6**, 5. (10.1186/1471-2164-6-5) PMC546002 15651988

[94] Ables ET, Bois KE, Garcia CA, Drummond-Barbosa D. 2015 Ecdysone response gene E78 controls ovarian germline stem cell niche formation and follicle survival in Drosophila. Dev. Biol. **400**, 33–42. (10.1016/j.ydbio.2015.01.013) 25624267 PMC4448935

[95] Clifton ME, Noriega FG. 2011 Nutrient limitation results in juvenile hormone-mediated resorption of previtellogenic ovarian follicles in mosquitoes. J. Insect Physiol. **57**, 1274–1281. (10.1016/j.jinsphys.2011.06.002) 21708165 PMC3167010

[96] Clifton ME, Noriega FG. 2012 The fate of follicles after a blood meal is dependent on previtellogenic nutrition and juvenile hormone in Aedes aegypti. J. Insect Physiol. **58**, 1007–1019. (10.1016/j.jinsphys.2012.05.005) 22626792 PMC3389259

[97] Ahmed AM, Hurd H. 2006 Immune stimulation and malaria infection impose reproductive costs in Anopheles gambiae via follicular apoptosis. Microbes Infect. **8**, 308–315. (10.1016/j.micinf.2005.06.026) 16213176

[98] Uchida K, Ohmori D, Ueno T, Nishizuka M, Eshita Y, Fukunaga A, Kominami E. 2001 Preoviposition activation of cathepsin-like proteinases in degenerating ovarian follicles of the mosquito Culex pipiens pallens. Dev. Biol. **237**, 68–78. (10.1006/dbio.2001.0357) 11518506

[99] Moura AS, Cardoso AF, Costa-da-Silva AL, Winter CE, Bijovsky AT. 2015 Two cathepsins B are responsible for the yolk protein hydrolysis in Culex quinquefasciatus. PLoS One **10**, e0118736. (10.1371/journal.pone.0118736) 25710877 PMC4339980

[100] Zieler H, Dvorak JA. 2000 Invasion in vitro of mosquito midgut cells by the malaria parasite proceeds by a conserved mechanism and results in death of the invaded midgut cells. Proc. Natl Acad. Sci. USA **97**, 11516–11521. (10.1073/pnas.97.21.11516) 11027351 PMC17232

[101] Ramelow J, Keleta Y, Niu G, Wang X, Li J. 2023 Plasmodium parasitophorous vacuole membrane protein Pfs16 promotes malaria transmission by silencing mosquito immunity. J. Biol. Chem. **299**, 104824. (10.1016/j.jbc.2023.104824) 37196765 PMC10276155

[102] Ramphul UN, Garver LS, Molina-Cruz A, Canepa GE, Barillas-Mury C. 2015 Plasmodium falciparum evades mosquito immunity by disrupting JNK-mediated apoptosis of invaded midgut cells. Proc. Natl Acad. Sci. USA **112**, 1273–1280. (10.1073/pnas.1423586112) 25552553 PMC4321252

[103] Klug D, Arnold K, Mela-Lopez R, Marois E, Blandin SA. 2022 A toolbox of engineered mosquito lines to study salivary gland biology and malaria transmission. PLoS Pathog. **18**, e1010881. (10.1371/journal.ppat.1010881) 36223382 PMC9555648

[104] Arora G, Chuang YM, Sinnis P, Dimopoulos G, Fikrig E. 2023 Malaria: influence of Anopheles mosquito saliva on Plasmodium infection. Trends Immunol. **44**, 256–265. (10.1016/j.it.2023.02.005) 36964020 PMC10074230

[105] Martin-Martin I *et al*. 2020 ADP binding by the Culex quinquefasciatus mosquito D7 salivary protein enhances blood feeding on mammals. Nat. Commun. **11**, 2911. (10.1038/s41467-020-16665-z) 32518308 PMC7283271

[106] Cator LJ, Lynch PA, Read AF, Thomas MB. 2012 Do malaria parasites manipulate mosquitoes? Trends Parasitol. **28**, 466–470. (10.1016/j.pt.2012.08.004) 23044288 PMC3478439

[107] Stanczyk NM *et al*. 2019 Species-specific alterations in Anopheles mosquito olfactory responses caused by Plasmodium infection. Sci. Rep. **9**, 3396. (10.1038/s41598-019-40074-y) 30833618 PMC6399344

[108] Koella JC, Sørensen FL, Anderson RA, Flemming L, Sørensen L. 1998 The malaria parasite, Plasmodium falciparum increases the frequency of multiple feeding of its mosquito vector, Anopheles gambiae. Proc. R. Soc. B **265**, 763–768. (10.1098/rspb.1998.0358) PMC1689045 9628035

[109] Smallegange RC, van Gemert GJ, van de Vegte-Bolmer M, Gezan S, Takken W, Sauerwein RW, Logan JG. 2013 Malaria infected mosquitoes express enhanced attraction to human odor. PLoS One **8**, e63602. (10.1371/journal.pone.0063602) 23691073 PMC3655188

[110] Cator LJ, George J, Blanford S, Murdock CC, Baker TC, Read AF, Thomas MB. 2013 ‘Manipulation’ without the parasite: altered feeding behaviour of mosquitoes is not dependent on infection with malaria parasites. Proc. R. Soc. B **280**, 20130711. (10.1098/rspb.2013.0711) PMC3774228 23698008

[111] Vantaux A, de Sales Hien DF, Yameogo B, Dabiré KR, Thomas F, Cohuet A, Lefèvre T. 2015 Host-seeking behaviors of mosquitoes experimentally infected with sympatric field isolates of the human malaria parasite Plasmodium falciparum: no evidence for host manipulation. Front. Ecol. Evol. **3**, 86. (10.3389/fevo.2015.00086)

[112] Hogg JC, Hurd H. 1995 Malaria‐induced reduction of fecundity during the first gonotrophic cycle of Anopheles Stephensi mosquitoes. Med. Vet. Entomol. **9**, 176–180. (10.1111/j.1365-2915.1995.tb00175.x) 7787226

[113] Ferguson HM, Rivero A, Read AF. 2003 The influence of malaria parasite genetic diversity and anaemia on mosquito feeding and fecundity. Parasitology **127**, 9–19. (10.1017/s0031182003003287) 12885184

[114] Cozzarolo CS, Glaizot O, Christe P, Pigeault R. 2020 Enhanced attraction of arthropod vectors to infected vertebrates: a review of empirical evidence. Front. Ecol. Evol. **8**, 568140. (10.3389/fevo.2020.568140)

[115] Kefi M, Cardoso-Jaime V, Saab SA, Dimopoulos G. 2024 Curing mosquitoes with genetic approaches for malaria control. Trends Parasitol. **40**, 487–499. (10.1016/j.pt.2024.04.010) 38760256

[116] Garcia-Longoria L, Berthomieu A, Hellgren O, Rivero A. 2025 Supplementary material from: Avian malaria and the overlooked metabolic pathways underlying mosquito-Plasmodium interactions. FigShare. (10.6084/m9.figshare.c.8086216) PMC12539972 41127797

